# p53 mutation in normal esophagus promotes multiple stages of carcinogenesis but is constrained by clonal competition

**DOI:** 10.1038/s41467-022-33945-y

**Published:** 2022-10-20

**Authors:** Kasumi Murai, Stefan Dentro, Swee Hoe Ong, Roshan Sood, David Fernandez-Antoran, Albert Herms, Vasiliki Kostiou, Irina Abnizova, Benjamin A. Hall, Moritz Gerstung, Philip H. Jones

**Affiliations:** 1 Wellcome Sanger Institute, Hinxton, CB10 1SA United Kingdom; 2 European Molecular Biology Laboratory, European Bioinformatics Institute, Cambridge, CB10 1SD United Kingdom; 3 Department of Medical Physics and Biomedical Engineering, University College London, London, United Kingdom; 4 Department of Oncology, University of Cambridge, Cambridge, UK; 5 Present Address: DKFZ, Im Neuenheimer Feld 280, 69120 Heidelberg, Germany; 6 Present Address: Wellcome/Cancer Research UK Gurdon Institute, Henry Wellcome Building of Cancer and Developmental Biology, Tennis Court Road, Cambridge, CB2 1QN United Kingdom

**Keywords:** Oncogenesis, Oesophageal cancer, Cancer genomics

## Abstract

Aging normal human oesophagus accumulates *TP53* mutant clones. These are the origin of most oesophageal squamous carcinomas, in which biallelic *TP53* disruption is almost universal. However, how *p53* mutant clones expand and contribute to cancer development is unclear. Here we show that inducing the *p53*^*R245W*^ mutant in single oesophageal progenitor cells in transgenic mice confers a proliferative advantage and clonal expansion but does not disrupt normal epithelial structure. Loss of the remaining *p53* allele in mutant cells results in genomically unstable *p53*^*R245W/null*^ epithelium with giant polyaneuploid cells and copy number altered clones. In carcinogenesis, *p53* mutation does not initiate tumour formation, but tumours developing from areas with *p53* mutation and LOH are larger and show extensive chromosomal instability compared to lesions arising in wild type epithelium. We conclude that *p53* has distinct functions at different stages of carcinogenesis and that LOH within *p53* mutant clones in normal epithelium is a critical step in malignant transformation.

## Abstract (web summary)

Ageing normal oesophagus epithelium contains p53 mutant clones. Here the authors use transgenic mice to show how these clones form and contribute to cancer development.

## Introduction

As humans age, our normal tissues become populated by mutant clones under strong positive selection, including genes recurrently mutated in cancer^1–9^. A typical example is the progressive increase in prevalence of clones carrying heterozygous *TP53* mutants in normal oesophagus, which reaches 5–10% of the tissue by middle age rising to 15–30% for those in their 70s^2,5^. Despite the accumulation of *TP53* mutants, human oesophagus remains histologically normal and genome stability is maintained other than frequent LOH at the *NOTCH1* locus^2^. Indeed, given the prevalence of *TP53* mutations the incidence of cancer seems surprisingly low.

**Fig. 1:**
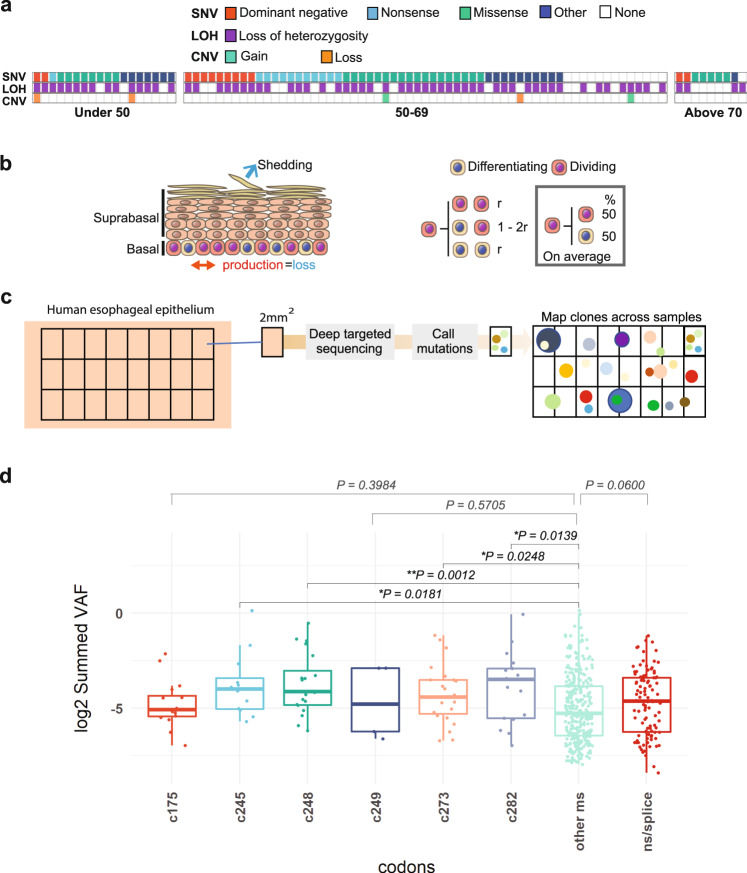
*TP53* mutation in Oesophageal Squamous Cell Carcinoma (ESCC). **a** 88 TCGA ESCC samples were analyzed to identify the prevalence of genome alterations (single nucleotide variants, SNV, loss of heterozygosity, LOH & copy number variation, CNV) in the *TP53* gene. 74% of samples reported LOH over *TP53* and 84% reported SNVs. 64% of samples reported both SNVs and LOH over *TP53*. See Supplementary Data 1 for source data. **b** Cell behaviour in basal layer of normal homoeostatic epithelium. On division, a progenitor may generate two dividing progenitors, two differentiating daughters, or one cell of each type. *r* is the probability of symmetric division outcome. On average, equal proportions of progenitor and differentiating cells are generated. **c**, **d** *TP53* mutant clones in normal human oesophagus, data from ref. 2. **c** Protocol: normal oesophageal epithelium was cut into a contiguous grid of 2 mm^2^ samples and ultradeep targeted sequencing of 74 cancer genes, including *TP53*, performed. SNV were called and those spanning adjacent samples merged to generate the variant allele frequency (VAF) for each SNV. **d** VAF of *TP53* mutants. VAFs of missense mutants at codons 175, 245, 248, 249 273, and 282 are compared with the other missense (other ms) and nonsense and essential splice mutants (ns/splice). *n* = 16, 12, 20, 5, 23, 17, 307, and 101 clones respectively. Boxes indicate quartiles, horizontal bar median and whiskers indicate range, up to 1.5 fold inter-quartile range. *p* values, Kruskal–Wallis test for between group differences with Dunnett’s correction for multiple comparisons. See Supplementary Data 2.

In contrast to normal tissue, sequencing of squamous cell carcinoma (SCC) shows that the majority carry protein altering *TP53* mutations, most of which are associated with LOH^10,11^ (Fig. 1a). Multiregional sequencing indicates biallelic *TP53* disruption is a truncal event in SCC^10^. SCC are characterized by marked genomic instability, consistent with prior *TP53* loss being permissive of the survival of whole chromosomal aneuploid cells^11,12^. Taken together, these results suggest *TP53* loss is required for SCC development and that tumours may originate via the rare clones with biallelic *TP53* disruption among the heterozygous *TP53* mutant population in normal tissue^2^.

These observations motivated us to investigate the roles of *Trp53 (p53*) mutation on cell dynamics and clonal fitness in oesophageal carcinogenesis using a transgenic mouse model.

Normal mouse oesophagus has a simple, uniform structure that makes it ideal for studying the effects of mutations on cell behaviour. The tissue consists of layers of keratinocytes. Dividing cells lie in the deepest, basal cell layer and are a single population of equipotential progenitor cells. Progenitor divisions generate either two progenitor daughters, two differentiating cells or one cell of each type (Fig. 1b). The likelihood of each division outcome is balanced so that one progenitor and one differentiating daughter are produced from an average division. It follows that equal numbers of progenitor and differentiating cells are generated across the progenitor population, thereby maintaining tissue homoeostasis. Differentiating cells exit the cell cycle and leave the basal layer migrating to the tissue surface from which they are shed (Fig. 1b). Cells are continuously shed and replaced throughout life.

In transgenic mice it is possible to track the fate of cohorts of mutant cells in adult animals by lineage tracing. Scattered single progenitor cells are induced to express a mutation and a fluorescent reporter protein by genetic recombination. The daughter cells of the mutant progenitor may be visualized by 3D imaging and quantifying the proliferating and differentiating cells in clones provides insight into wild type and mutant cell dynamics^13–15^.

In this work, we apply lineage tracing in a transgenic mouse model to study how heterozygous *p53* mutant clones colonized the normal oesophagus, the effects of loss of the remaining wild type *p53* allele on clonal fitness and genome stability, and the role(s) of *p53* mutants in tumour development. We find that oesophageal progenitors expressing a heterozygous p53 mutant have a proliferative advantage over wild type progenitors by virtue of a bias in cell fate. On average, mutant cells generate more progenitor than differentiating daughters at each round of cell division, thus outcompeting wild type progenitors that produce equal proportions of progenitor and differentiating cells. Despite this, the heterozygous epithelium remains phenotypically normal. However, loss of the second *p53* allele in *p53* mutant cells results in the appearance of giant polyaneuploid cells and the emergence of copy number altered clones. In carcinogenesis experiments, tumours arising from epithelium in which both alleles of *p53* are disrupted are larger and show extensive copy number alterations (CNAs). We conclude that heterozygous p53 mutation is sufficient to drive clonal expansion, but the loss of the second *p53* allele is required to acquire a copy number unstable phenotype and promote tumour growth.

## Results

### *TP53* mutant clonal expansions in humans

We began by analyzing data from a study that mapped *TP53* mutant clones in human oesophagus by deep sequencing arrayed samples of epithelium (Fig. 1c)^2^. Clones that spanned adjacent samples were merged and the proportion of sequencing reads (variant allele frequency, VAF) carrying each single nucleotide variant calculated. As CNAs at the *TP53* locus were detected only rarely (in 0.4% of clones), clone size may be assumed to be proportional to VAF^2^. The distribution of VAFs was plotted for missense *TP53* mutants at the frequently mutated ‘hotspot’ codons 175, 245, 248, 249, 273, and 282 in the DNA binding domain of the protein, the remaining *TP53* missense mutants, and nonsense and essential splice mutants that result in a null allele^16,17^. There was no significant difference between the VAF distributions of the nonsense/essential splice and non-hotspot mutants (*P* = 0.06, Kruksal–Wallis test, *n* = 307 and 101 clones respectively). However, the VAFs of missense mutants at codons 245, 248, 273, and 282 were significantly higher than the non-dominant negative missense mutants (*p* = 0.018, 0.0012, 0.025, and 0.014, *n* = 12, 20, 23, 17, and 307 clones respectively, Kruskal-Wallis test for between-group differences with Dunnett’s correction for multiple comparisons, Fig. 1d). These findings suggest that in humans, clones carrying codon 248, 245, 273, and 282 dominant-negative *TP53* missense mutants may have increased competitive fitness relative to missense mutations at other codons evidenced by their potential to drive clonal expansion in the oesophagus. Motivated by this possibility, we investigated the role of the murine equivalent of a codon 248 mutant, *TP53*^*R248W*^, that occurs commonly both in clonal expansions in normal oesophagus and in oesophageal SCC (https://cancer.sanger.ac.uk/cosmic/^2^).

### Heterozygous *p53*^*R245W*^ mutation drives mutant clone expansion in murine oesophagus

**Fig. 2:**
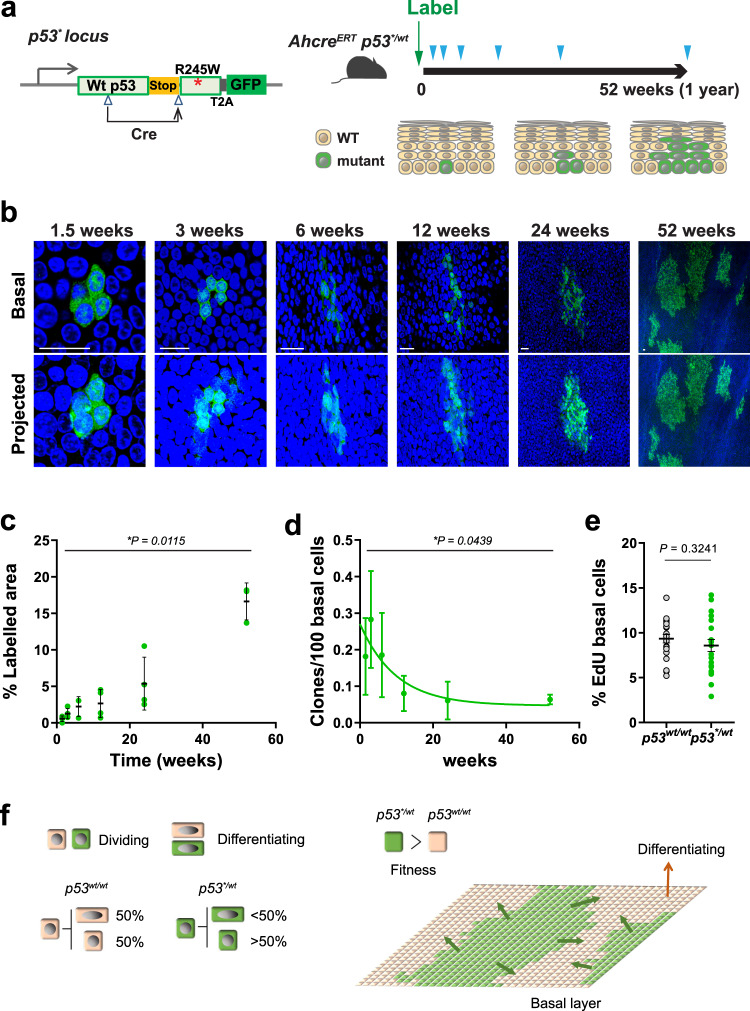
Heterozygous *p53*^*R245W*^ (p53^*/wt^) mutant cell fate in mouse oesophagus. **a** Protocol and schematic of genetic lineage tracing in *Ahcre*^*ERT*^ *p53* ^*flR245W-GFP/wt*^ (p53^*/wt^) mice. Expression of the *p53* mutant allele and GFP reporter was induced in scattered single cells (Labelling) and oesophagus samples were taken at indicated time points (triangles). The fate of mutant clones was examined by tracking the expression of GFP. **b**–**d** *n* = 4 mice per time point except *n* = 3 at the 6-week and 52-week time points, from 3 independent experiments. **b** Rendered confocal z stacks showing typical p53^*/wt^ clones in oesophageal epithelial wholemounts. Basal, top-down view of basal layer, Projected, top-down view through all nucleated cell layers. Green, GFP; blue, DAPI; Scale bars, 20 µm **c** Proportion of projected area labelled with GFP at indicated time points. Average value from 6 fields per animal. Error bars are mean ± s.e.m. **d** Density of p53^*/wt^ clones over the time. Error bars are mean ± s.e.m. For **c**, **d**, *p* value was determined by Kruskal–Wallis test. **e** Average percentage of EdU-positive basal cells in p53^*/wt^ clones compared to non-GFP labelled (*p53*^*wt/wt*^) basal cells in the same mouse. EdU was administered an hour before sampling. *n* = 22 mice across all time points. Error bars are mean ± s.e.m. *p* value, two-tailed paired Student’s t test. See Supplementary Data 5–7. **f** Schematic illustration of p53^*/wt^ cell behaviour. On average, wild type progenitors (*p53*^*wt/wt*^) produce equal proportions of dividing and differentiating cells across the population, whereas p53^*/wt^ cells generate more dividing cells. This fate imbalance allows p53^*/wt^ cells to outcompete wild type cells and expand in the tissue.

To confirm whether heterozygous dominant negative missense *p53* mutation alters the behaviour of oesophageal progenitors we turned to a transgenic mouse model. In *Ahcre*^*ERT*^*p53*^*flR245W-GFP/wt*^ mice, hereafter termed p53^*/wt^, *Trp53*^*R245W*^, corresponding to human *TP53*^*R248W*^, can be inducibly expressed following a genetic recombination event triggered by a drug regulated form of *cre* recombinase (Fig. 2a)^18,19^. The p53^*^ mutant is functionally different from a null allele and is frequently detected in squamous and other cancers^20,21^. Significantly, in human oesophagus, R248W and R248Q mutants show no difference in clonal VAF (*p* = 0.73, Mann–Whitney Test). Mutant cells can be tracked as an expression of the mutant is linked to a green fluorescent protein (GFP) reporter. As a control, we tracked the behaviour of *p53*^*wt/wt*^ progenitors in the *Ahcre*^*ERT*^*Rosa26*^*flYFFP/wt*^ (abbreviated to R^YFP^) mouse strain, where wild type cells are genetically labelled with a functionally neutral yellow fluorescent protein (YFP) reporter^14^.

Heterozygous p53* or control YFP expression was induced in scattered single cells following the induction of *cre* recombinase in a cohort of adult mice. Expression of the GFP or YFP reporter is inherited by the progeny of the labelled cell, resulting in the formation of clones (Fig. 2a and Supplementary Fig. 1a). We prepared wholemounts of oesophageal epithelium at different time points after induction, immunostained for the reporter protein and then performed 3D imaging of the tissue at single cell resolution using confocal microscopy. The number and location of cells in an unselected sample of clones was determined at each time point.

In *p53*^*wt/wt*^, R^YFP^ mice, the number of clones declined with time. This is due to differentiation, as if all the proliferating cells in a clone differentiate, the clone leaves the basal cell layer, migrates to the epithelial surface, and is shed from the tissue^14^. However, the size of the remaining clones rose progressively, with the net result that the proportion of labelled cells in the epithelium remained constant over time (Supplementary Data 3, 4). Such behaviour is indicative of neutral competition between labelled and unlabelled wild type functionally equivalent progenitor cells (Supplementary Fig. 1b, c)^14^.

By contrast, in induced p53^*/wt^ mice the area of labelled epithelium rose progressively indicating the mutant population had a competitive advantage over wild type neighbours (Fig. 2b, c). We found that GFP expression diminished in the upper suprabasal layers close to the epithelial surface, reflecting decreased transcription at the *p53* locus in differentiating cells^19^. To visualize mutant clones in all layers of the epithelium, we crossed *Ahcre*^*ERT*^p53^*/wt^ mice with the *Rosa26*^*Confetti/wt*^ reporter strain and induced recombination in single progenitors. This generated clones expressing red fluorescent protein from the constitutive *Rosa26* locus as well as GFP, and showed that differentiating p53^*/wt^ cells extend through the upper differentiating layers of the tissue and reach the surface of epithelium, confirming mutant cells can be lost through shedding (Supplementary Fig. 1d, e)^19,22^.

We next investigated the cellular mechanism(s) of p53^*/wt^ clonal expansion. The mean number of basal layer cells/clone was consistently higher in p53^*/wt^ clones than in YFP expressing clones in R^YFP^ mice at the corresponding time point (*p* < 0.001 at 3, 6, and 12 weeks, two-tailed Mann–Whitney test, Supplementary Fig. 1b). The number of labelled mutant clones decreased over time consistent with mutant clone loss through differentiation (Fig. 2d). Further, the proportion of basal cells in S phase of the cell cycle labelled with a pulse of 5-ethynyl-2′-deoxyuridine (EdU) was similar in mutant and wild type cells, suggesting that the rate of cell division and proportion of proliferating cells were not substantially altered by the mutation (Fig. 2e). A lack of change in cell division rate is also seen with other mutations that colonize the oesophagus, and may reflect the requirement for a nearby differentiating cell to exit the basal layer before a cell can divide in squamous epithelia^19,23–25^.

Taken together, these observations may be explained by a model in which p53^*/wt^ progenitors generate more progenitor than differentiating daughters per average cell division, so that the population grows despite no change in the rate of mutant cell division from that of wild type cells. To test this hypothesis, we explored a simple quantitative simulation of wild type and mutant cell dynamics, a two-dimensional stochastic cellular automaton (CA) model (Supplementary Fig. 2). Mutant cells had a bias in cell fate, with an increased likelihood of dividing to generate two progenitor daughters compared with wild type cells which had balanced cell fate. This proliferative advantage decreased when local cell crowding occurred, reflecting the spatial constraints of normal epithelium. This model gave a good quantitative fit to the observed clone sizes in vivo for both wild type and mutant cells (Supplementary Fig. 2b). We conclude that a bias in p53^*/wt^ mutant progenitor cell fate towards proliferation with no change in division rate is consistent with the clonal dynamics observed in lineage tracing.

To gain further insight into the phenotype of p53^*/wt^ epithelium, we induced *Ahcre*^*ERT*^p53^*/wt^ mice with a higher dose of inducing agents to yield at a higher initial clone density and aged them for a year, by which time almost 70% of the epithelium was colonized by p53^*/wt^ cells (Supplementary Fig. 3). The tissue remained macroscopically normal with no visible tumours. There was no apparent difference in histology or immunostaining for the marker of basal cells, KRT14 and the protein LOR, expressed in differentiating cells, comparing p53^*/wt^ and wild type epithelium (Supplementary Fig. 3). Furthermore, there was no significant difference in the density of CD45 positive leucocytes in the p53* expressing epithelium (Supplementary Fig. 3e, f). Thus, as in humans, tissue integrity is maintained despite the presence of heterozygous *p53* mutants.

We conclude that cells carrying a heterozygous missense *p53* mutation colonize normal epithelium due to a subtle bias in progenitor fate towards proliferation (Fig. 2f). This results in a slowly expanding mutant population that will persist long term in the tissue, with the potential to undergo further genomic alterations.

### Loss of the second *p53* allele does not increase the competitive fitness of p53* mutant cells

**Fig. 3:**
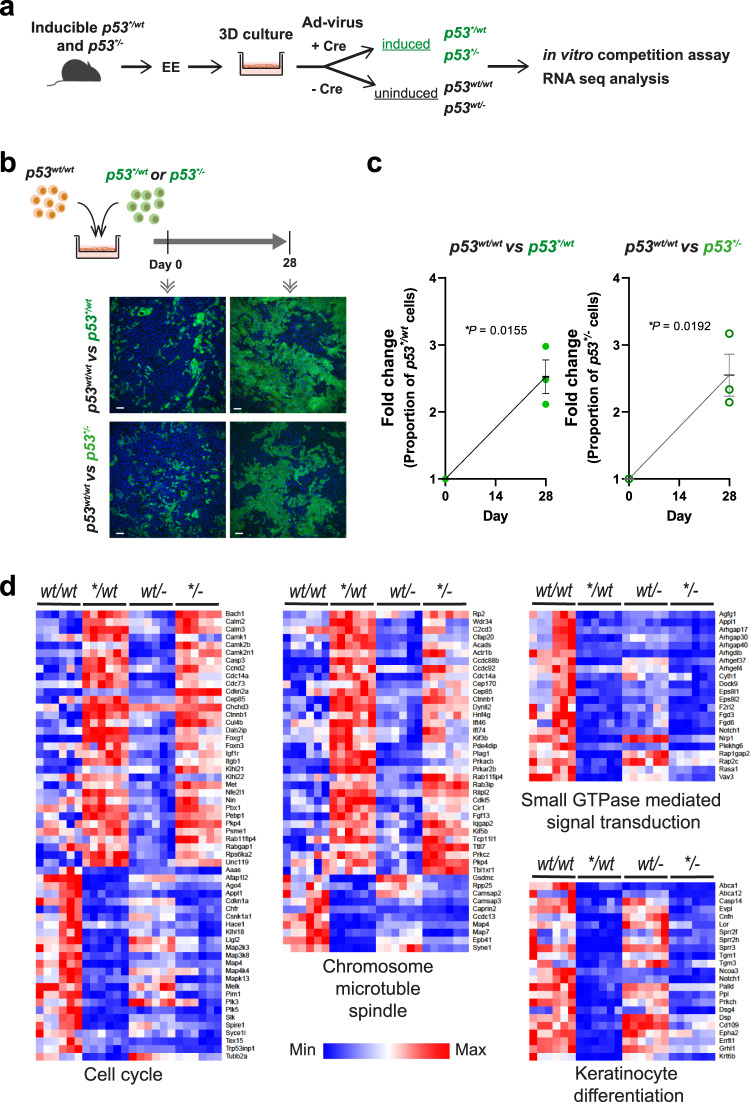
Effect of loss of heterozygosity (LOH) on mutant cell behaviour. **a** A 3D primary culture system was used to characterize *p53* mutant cells in vitro. Oesophageal keratinocytes were isolated from transgenic mice and p53* mutation was induced by adenovirus carrying cre recombinase. **b** Cell competition assay. p53^*/wt^ and p53^*/−^ cells were co-cultured with *p53*^*wt/wt*^ cells respectively and relative fitness was examined. Representative immunofluorescence images from three biological replicates at day 0 and 28 are shown. Green, GFP; blue, DAPI. Scale bars, 50 µm. **c** Quantitation of cell competition assay by flow cytometry. Graph shows the fold change of proportion of GFP + p53^*/wt^ or p53^*/−^ cell in the culture. Black lines indicate mean and s.e.m. *p* value, two-tailed ratio paired t-test, *n* = 3 replicate cultures. **d** RNAseq analysis showing differentially expressed transcripts in p53 mutant cells which could affect the fitness of cells. RNAseq data were subjected to the Gene Ontology analysis (enrichGO in R package clusterProfiler). Enriched biological processes in pairwise of comparisons both p53^*/wt^ vs *p53*^*wt/wt*^ and p53^*/−^ vs *p53*^*wt/−*^ were considered to be affected by p53* expression. Heatmaps were generated for genes associated with the cell cycle, cell division (chromosome, microtubule, and spindle), small GTPase mediated signal transduction, and keratinocyte differentiation. *n* = 6 biological replicates per genotype. See Supplementary Data 8–11.

The fitness of some heterozygous mutant genes that colonize the normal oesophagus, such as *Notch1*, is increased following loss of the second allele, enhancing colonization and creating a selecting pressure towards LOH^25^. To determine whether this was the case with p53^*/wt^ cells we turned to a competition assay in organotypic culture (Fig. 3a)^18^. Wild type cells were co-cultured for 4 weeks with either p53^*/wt^ or p53^*/−^ cells. Both mutant genotypes outcompeted WT cells to a similar degree. This indicates that p53* acts as a dominant negative for competitive fitness and hence that LOH confers no additional fitness advantage on p53^*/wt^ cells (Fig. 3b, c). This observation has important implications, as if p53^*/wt^ cells develop LOH they will compete neutrally with their p53^*/wt^ neighbours, and the majority of the biallelic mutant clones may be lost through neutral drift rather than persisting to acquire further genomic alterations.

To gain more insight into the effects of p53* expression on cell states we analyzed the transcriptome in organotypic cultures of *p53*^*wt/wt*^, p53^*/wt^, *p53*^*wt/−*^ and p53^*/−^ oesophageal epithelium. RNA sequencing identified transcripts differentially expressed in p53^*/wt^ and p53^*/−^ compared with *p53*^*wt/wt*^ and *p53*^*wt/−*^ cultures respectively (Fig. 3a, d and Supplementary Fig. 4a). Expression of p53* with either a wild type or null *p53* allele enriched biological processes from Gene Ontology analysis included proliferation/cell cycle, keratinocyte differentiation, small GTPase signal transduction as well as metabolic and apoptotic processes known to be regulated by *p53*^18,26^. The expression of multiple keratinocyte differentiation markers was downregulated and the expression of cell cycle genes was altered in both p53^*/wt^ and p53^*/−^ compared to *p53*^*wt/wt*^ and *p53*^*wt/−*^ cells (Fig. 3d and Supplementary Data 10, 11). Regulators of cytokinesis including microtubular, mitotic spindle, and small GTPase-mediated signal transduction genes also perturbed. These results suggest that p53* mutant expression with or without a second wild type *p53* allele leads to mis-regulation of multiple transcriptional programmes that may impact competitive fitness.

### p53^*/−^ epithelium

**Fig. 4:**
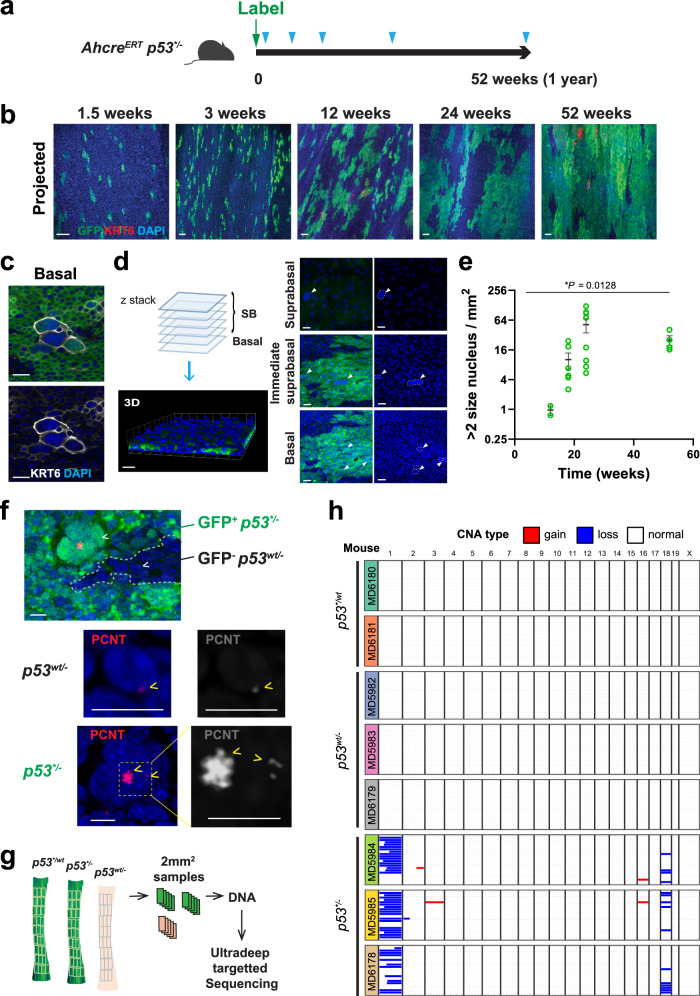
p53^*/−^ oesophageal epithelium. **a** Protocol: p53^*/−^ mice were generated with an inducible p53* allele and a *p53* null allele. Following induction, p53^*/−^ clones competed in a *p53*^*wt/−*^ background, and were sampled at indicated time points (triangles). **b**–**d** Images are representative from *n* = 2 mice for 1.5 and 3 week, *n* = 4 for 12, 24, and 52 week time points. **b** Rendered confocal z-stacks showing projected views of p53^*−^ clones in wholemounts. Images are representative from Green, GFP; red, KRT6; blue, DAPI; Scale bars, 40 µm. **c** Confocal images showing cells with aneuploid appearance in p53^*/−^ clone area. Green, GFP; grey, KRT6; blue, DAPI; Scale bars, 20 µm. **d** Confocal z-stack images were used to reconstruct a 3D image of the wholemount sample and determine the z-position of cells of interest. Aneuploid-like cells were found both in the basal and suprabasal layers. Scale bars, 20 µm. **e** Number of cells with large (≥double size) nucleus in p53^*/−^ clone area post induction. *n* = 2 mice for 12 week time point, *n* = 6 for 18 week, *n* = 8 for 24 week and *n* = 4 for 52 week time points. Error bars are mean ± s.e.m. *p* value determined by Welch’s ANOVA. See Supplementary Data 13. **f** Representative images of above samples stained for PCNT. *n* = 4 mice. GFP-positive p53^*/−^ cells (green) and GFP negative *p53*^*wt/−*^ cells are from the same EE. Grey dashed line indicates the border of GFP+ and GFP− area. Each representative nucleus (arrowheads) were shown at higher magnification. PCNT foci (yellow arrowheads) were also shown in grey scale on the right. For p53^*/−^ samples, the yellow dashed box is shown at higher magnification. Red, PCNT; green, GFP; blue, DAPI. Scale bars, 10 µm. **g** Wholemount epithelium (for protocol, see Supplementary Fig. 5a) was cut into a contiguous grid of 2 mm^2^ pieces, DNA was extracted and subjected to ultradeep targeted sequencing. **h** Summary of copy number analysis using targeted sequencing data. Further analysis with Low coverage WGS from same experiment is shown in Supplementary Fig. 5f.

The genotype of SCC argues that they develop from the biallelic *p53* mutant clones that do succeed in persisting in the epithelium, so we set out to characterize the p53^*/−^ phenotype. *Ahcre*^*ERT*^p53^*/wt^ mice were crossed with a *p53*^*−/−*^ strain. When the resulting *Ahcre*^*ERT*^p53^*/−^ animals were induced, GFP expressing p53^*/−^ clones competed in a background of unlabelled *p53*^*wt/−*^ cells (Fig. 4a, b). The area occupied by p53^*/−^ cells increased rapidly so that by a year almost the entire epithelium was replaced by p53^*/−^ mutant cells, indicating they were substantially fitter than their *p53*^*wt/−*^ neighbours. Consistent results were seen when p53^*/−^ cells were co-cultured with *p53*^*wt/−*^ cells (Supplementary Fig. 4c–f).

In terms of phenotype, p53^*/−^ epithelium looked macroscopically normal, tissue integrity was maintained and no tumours developed. Histology and the expression of KRT14 and LOR appeared unaltered compared with *p53*^*wt/wt*^ and *p53*^*wt/−*^ tissue, and there was no change in the density of CD45 positive cells (Supplementary Fig. 3). However, confocal imaging of epithelial wholemounts revealed the progressive accumulation of scattered giant cells with enlarged nuclei in the GFP+ p53^*/−^ areas but not the adjacent *p53*^*wt/−*^ regions. These abnormal cells expressed the keratinocyte stress marker KRT6 and were detected both in the basal layer and the suprabasal layers (28.1% ± 4.2 in the suprabasal layer, *n* = 6 from 24 to 52 week time points), arguing they can be lost by differentiation and shedding (Fig. 4b–d). The early accumulation of cells with large nuclei indicates these cells were initially generated at a faster rate than they were shed. At later time points, however, the number of these abnormal cells levelled off, indicating a balance between generation and shedding had been established (Fig. 4e). Cytologically, the cells have features of giant polyaneuploid cells described in tumours and cell lines placed under genotoxic stress^27,28^.

We speculated that the emergence of nuclear enlargement may indicate the development of genomic instability in the p53^*/−^ epithelium. To investigate this, we first investigated the number of centrosomes in the cells with enlarged nuclei, as centrosomal amplification results in aneuploidy and tumorigenesis^29^. Staining for the centrosomal protein PCNT, pericentrin, revealed large or multiple foci consistent with centrosome amplification in the abnormal cells (82%, 42 out of 51 polyaneuploid nuclei) (Fig. 4f). We concluded that in p53^*/−^ epithelium, cells with centrosomal amplification develop and persist. Thus, biallelic p53 disruption appears permissive of aneuploidy.

### Aging of *p53* mutant epithelium

In humans, *p53* mutant clones in normal epithelium may persist for many years. We, therefore, investigated the effects of aging on p53* mutant epithelium on the mutational landscape and CNAs in oesophageal epithelium. Targeted sequencing for 142 genes associated with mouse and human epithelial cancers was performed on a gridded array of 2mm^2^ epithelial samples in oesophagus 1 year after induction of p53^*/wt^, *p53*^*wt/−*^ and p53^*/−^ mutants (Supplementary Fig. 5a, “Methods” and Supplementary Data 14, 15)^30^. Mean coverage exceeded 600× (Supplementary Data 14). Mutations were called using the Shearwater ML algorithm, which exploits the observed error rates per site in a large library of normal samples sequenced with the same bait set on the same sequencing platform to build a site-specific error model for every possible base change in all sequenced nucleotides. The median variant allele frequency of the 8747 SNV detected by *ShearwaterML* was 0.028 (Supplementary Data 14). Since the fraction of sequencing reads that carry a SNV is a function of the fraction of mutant cells within a sample and of the local copy number, we can integrate allele frequencies and sample area to obtain an approximate estimate of the size of detectable mutant clones. In cellular terms, the lower limit of detection is a clone of about 400 basal cells^30^.

We identified 26 unique somatic coding mutations in a total area of 80mm^2^ p53^*/wt^ epithelium (equivalent to 0.33 clones per mm^2^), and 50 and 46 mutations, each in total area 120 mm^2^ epithelium (0.41 and 0.38 mutant clones per mm^2)^ in *p53*^*wt/−*^ and p53^*/−^ oesophagus respectively (Supplementary Fig. 5b). The density of mutant clones in all three genotypes was similar to that in *p53*^*wt/wt*^ oesophagus (0.28 events per mm^2^)^30^. The mutational burden was also not significantly different between genotypes (Supplementary Fig. 5c). The functional impact of the mutations was also similar in p53* mutant and wildtype oesophagus (Supplementary Fig. 5d)^30^. Thus p53* mutation, with or without loss of the second *p53* allele, does not alter the incidence of age associated mutations in the normal mouse oesophagus.

We then looked for evidence of CNA in the aged epithelium using off-target sequencing reads. In p53^*/−^ tissue we observed recurrent loss of chromosome 1 and 18 in a small proportion of cells, consistent with clones with CNA within the samples (Fig. 4g, h). There was no evidence of such chromosomal instability in p53^*/wt^ and *p53*^*wt/−*^ epithelium. The calls were similar to those obtained from low coverage whole genome sequencing (Supplementary Fig. 5f). These results argue that *p53* mutation with LOH leads chromosomal instability.

### Dynamics of p53* mutant clones in mutagenized epithelium

Aging human oesophagus is a patchwork of mutant clones under strong competitive selection. To persist in such an environment *p53* mutant clones must outcompete neighbouring clones, which expand until they encounter clones of similar fitness^30^. Mice treated with diethylnitrosamine (DEN), a well characterized mutagen found in tobacco smoke, develop a similar mutational landscape to older humans^30^. In addition, DEN treatment followed by aging leads to the development of oesophageal tumours, both premalignant dysplasias and, more rarely, SCCs^30–32^.

**Fig. 5:**
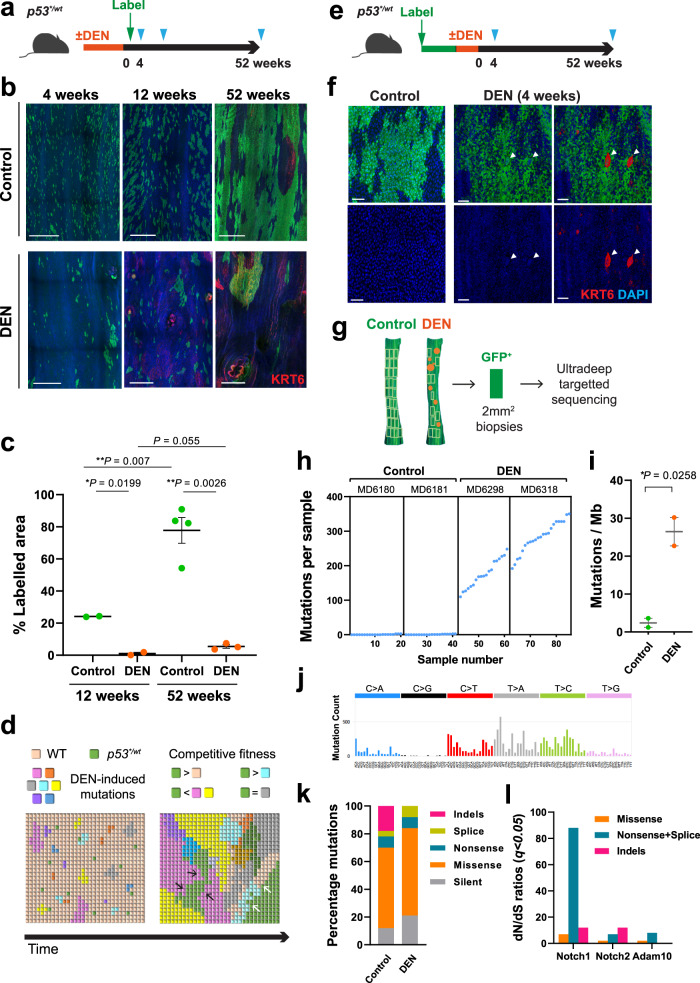
p53^*/wt^ mutant clones in mutagenized epithelium. **a** Protocol: p53^*/wt^ mice were treated with DEN or vehicle followed by clonal induction, oesophagus samples were collected at indicated time points (triangles). **b** Projected confocal z-stacks showing p53^*/wt^ clones in epithelial wholemounts. Representative images from two independent experiments. 4 weeks, *n* = 4 control and *n* = 3 DEN mice; 12 weeks, *n* = 2 mice per group; 52 weeks, *n* = 6 control and *n* = 4 DEN mice. Green, GFP; Red, KRT6; blue, DAPI. Scale bars, 500 µm. **c** Average proportion of projected labelled area. Number of mice as in **b**. Error bars, mean ± s.e.m., *p* values by two-tailed unpaired t-test with Welch’s t correction. See Supplementary Data 16. **d** Schematics of p53^*/wt^ clone behaviour in DEN-treated tissue. p53^*/wt^ outcompetes wild type cells, but not all DEN-induced mutant cells. Many p53^*/wt^ clones are lost in 4-weeks. Surviving p53^*/wt^ clones vary widely in size and are found in both epithelium and tumours. p53^*/wt^ clone can be outcompeted by surrounding fitter clones (black arrows) or itself be fitter than adjacent mutant clones (white arrows). **e** Protocol: Mice were treated with DEN or vehicle after colonization by p53^*/wt^ mutant cells. Triangles, sampling time points. **f** Top down views of basal layer of wholemounts from p53^*/wt^ induced mice 4 weeks after DEN treatment (*n* = 9 mice) compared to control (vehicle, *n* = 4 mice). Green, GFP; red, KRT6; blue, DAPI. Arrowheads indicate large nuclei in p53^*/wt^ clone area following DEN treatment. Scale bar, 50 µm. **g** Sequencing analysis at 1 year time point (protocol: **e**). DNA extracted from 2 mm^2^ grid biopsies were subjected to ultradeep targeted sequencing. Tumours (orange circles) were microdissected out from the epithelium prior to the following sequencing analysis. *n* = 40 (control) and 42 (DEN) biopsies from 2 mice per condition (**h**−**l**). **h** Number of mutations. Every dot corresponds to a sample. **i** Estimated mutation burden. *p* value by two-tailed unpaired Student’s t-test. Error bars, mean ± s.e.m. **j**, Mutational spectrum of DEN-treated p53^*/wt^ epithelium. **k** Percentage of mutation types identified in each condition. **l** Positively selected somatic mutations in DEN-treated samples. d*N*/d*S* ratios for missense, truncating (nonsense + splice) and indels. See Supplementary Data 14 and 15.

We began by studying how p53^*/wt^ mutant clones competed within a densely mutated normal epithelium. Mice were treated with DEN for 8 weeks, after which p53* expression was induced at a higher level than in a wild type background (Fig. 2) to allow for the elimination of p53^*/wt^ clones by DEN-induced mutants. The area occupied by p53* cells was monitored for up to a year post-induction (Fig. 5a). As expected, in controls which were not treated with DEN p53^*/wt^ mutant clones expanded, fused and occupied the majority of the epithelium over the year after induction (Fig. 5b, c). In contrast, the expansion of p53^*/wt^ mutant clones was severely reduced in the DEN-treated oesophageal epithelium, reaching only 5.5% of the area of the oesophagus by at the 52 week time point.

Both large and small or fragmented p53^*/wt^ clones were seen, consistent with clonal competition^19,30^ (Fig. 5b). Previous deep sequencing study identified numerous mutant clones in mouse oesophagus treated with same dose of DEN and found over 80% of epithelium was colonized by *Notch1* mutant clones 52 weeks after DEN treatment^30^. DEN also generates *p53* missense, nonsense, and essential splice mutant clones which are positively selected^30^. However, these are less competitive than *Notch1* mutants and occupy only about 5% of the epithelium 52 weeks post DEN, so have comparatively little impact on restricting p53*/^*wt*^ clonal expansion. Thus p53^*/wt^ clonal expansion is restricted by competing DEN-induced mutant clones such as *Notch1* in the epithelium (Fig. 5d)^30^. However, whilst their expansion is constrained, p53^*/wt^ mutant clones are sufficiently fit to persist in this highly competitive clonal landscape (Fig. 5b). These findings in mice recapitulate the restricted expansion and long-term persistence of heterozygous oncogenic *TP53* mutants in the highly competitive densely mutated normal epithelium of aging human oesophagus^2^.

Next, we performed an alternative protocol to model the effect of additional mutagenesis on p53^*/wt^ cells, as occurs within persisting *p53* mutant clones in humans. p53^*/wt^ mice were highly induced, the resulting clones allowed to expand, and the animals then treated DEN (Fig. 5e). Within 4 weeks after DEN treatment, cells expressing KRT6 with abnormally large nuclei were detected in GFP+ expressing areas, resembling those observed in p53^*/−^ epithelium, changes consistent with the emergence of aneuploid cells (Figs. 4 and 5f).

To further examine the effect on mutational landscape, we performed deep-targeted sequencing of the epithelium. This revealed 102 mutant clones/mm^2^ compared to ~2.4/mm^2^ in untreated p53^*/wt^ mice (Fig. 5g, h, “Methods”). The mutational burden was ~26 mutations per megabase in the DEN treated animals compared to ~2.4 in untreated p53^*/wt^ mice (Fig. 5i). Most mutations were protein altering and the mutation spectrum was dominated by T > A/A > T, T > C/A > G and C > T/G > A alterations (Fig. 5j, k), typical of DEN exposure^30^. To identify positively selected mutant genes in DEN-treated p53^*/wt^ epithelium, we calculated the dN/dS (non-synonymous-to synonymous mutation) ratios across all sequenced genes. Mutant *Notch1*, *Notch2,* and *Adam10* were positively selected in p53^*/wt^ epithelium (Fig. 5l). These results may be compared to those reported for *p53* wild type epithelium treated with the same protocol of DEN exposure^30^. The mutational burden is very similar and mutant genes under selection are unchanged in p53*^/wt^ and *p53*^*wt/wt*^ epithelium, indicating that p53* mutation does not alter the mutational landscape after DEN exposure.

Next, we explored the impact of the heterozygous p53^*/wt^ mutation on tumour formation, in two different protocols (Supplementary Fig. 6a). In protocol 1, mice were treated with DEN p53* mutation induced and animals aged, modelling the emergence of *p53* mutant clones within the already mutated landscape (Supplementary Fig. 6a, protocol 1). Persistent GFP + p53^*/wt^ clones were detected both within tumours but also in normal areas of EE at a year post induction (Fig. 5b). The 20% of lesions that contained GFP + p53* mutant cells were larger on average than the GFP negative tumours (Supplementary Fig. 6b). These observations argue the heterozygous p53* mutation does not initiate tumour formation but does promote tumour growth. In protocol 2, mice were induced and p53*clones allowed to expand, mice were then treated with DEN and aged, modelling the effect of additional mutations within *p53* mutant epithelium (Supplementary Fig. 6a). After 24 weeks, the number of tumours did not differ significantly between p53^*/wt^ induced and uninduced control (*p53*^*wt/wt*^) animals in either protocol (Supplementary Fig. 6c). The timing of p53^*/wt^ induction relative to DEN mutagenesis thus had no significant impact on the incidence of tumours. However, tumours originating from p53^*/wt^ epithelium were significantly larger than those in the uninduced controls (Supplementary Fig. 6a, d, protocol 2).

We then went on to characterize the p53^*/wt^ expressing lesions. Histology of macroscopic tumours showed the features of high-grade dysplasia, with loss of normal differentiation, architectural abnormalities, cell crowding, and loss of polarity (14 out of 16 tumours showed dysplasia or SCC) (Supplementary Data 21). In some cases, lesions extended deep into the submucosa (Supplementary Fig. 6e). Each of these features is also seen in tumours arising in DEN mutagenized epithelium in *p53*^*wt/wt*^ mice, although in a wild type background dysplastic lesions are smaller at the same time point and SCC appear later and are less frequent^33^. Two out of eleven p53^*/wt^ lesions developed into SCC by the 1 year time point (Supplementary Fig. 6f–i). Immunostaining of the SCC demonstrated a loss of expression of the terminal differentiation marker and cornified envelope precursor protein LOR. We also observed widespread expression of KRT14 which is normally confined to the basal layer and KRT6 which is a marker for murine premalignant and malignant oesophageal tumours^31^. In addition, immune cell infiltrates were detected by staining for the pan leucocyte marker, CD45. Staining for PCNT revealed multiple or clustered foci indicative of centrosome amplification (average 42% cells with >2 centrosomes (Supplementary Fig. 6j, k). This is significantly more frequent than in tumours arising from *p53*^*wt/wt*^ oesophagus where such PCNT foci occur in an average 15% of cells.

In parallel, we also examined the mutational landscapes of 11 of the larger tumours. For 5 lesions the adjacent epithelium was also sequenced. Deep targeted sequencing of 142 genes, with a mean on-target depth of coverage 183×, identified a total of 1373 mutations from 11 tumours and 722 from 5 adjacent epithelium (Supplementary Fig. 7a, b and “Methods”). The mutation burden and functional impact of mutations was similar between tumours and epithelium and the mutational spectrum was typical of DEN exposure (Supplementary Fig. 7c–e). Analysis of the variant allele frequency (VAF) indicated the epithelium was polyclonal whereas tumours other than SCCs were monoclonal (Supplementary Fig. 7f). In addition, within tumours, mutant *Notch1* and *Atp2a2* were positively selected (Supplementary Fig. 7g)^33^. We also looked for evidence of CNA in p53^*/wt^ tumours using off target reads of the above sequencing. 3 out of 12 macroscopic tumours and 2 out of 18 microscopic lesions had detectable CNA (Supplementary Fig. 7h, i), consistent with observation of a higher proportion of centrosome amplification in these tumours. This contrasts with DEN-induced tumours from *p53*^*wt/wt*^ mice, only 3% (2 of 64 tumours) exhibit CNA^33^.

### Effect of mutagenesis on p53^*/−^ epithelium

**Fig. 6:**
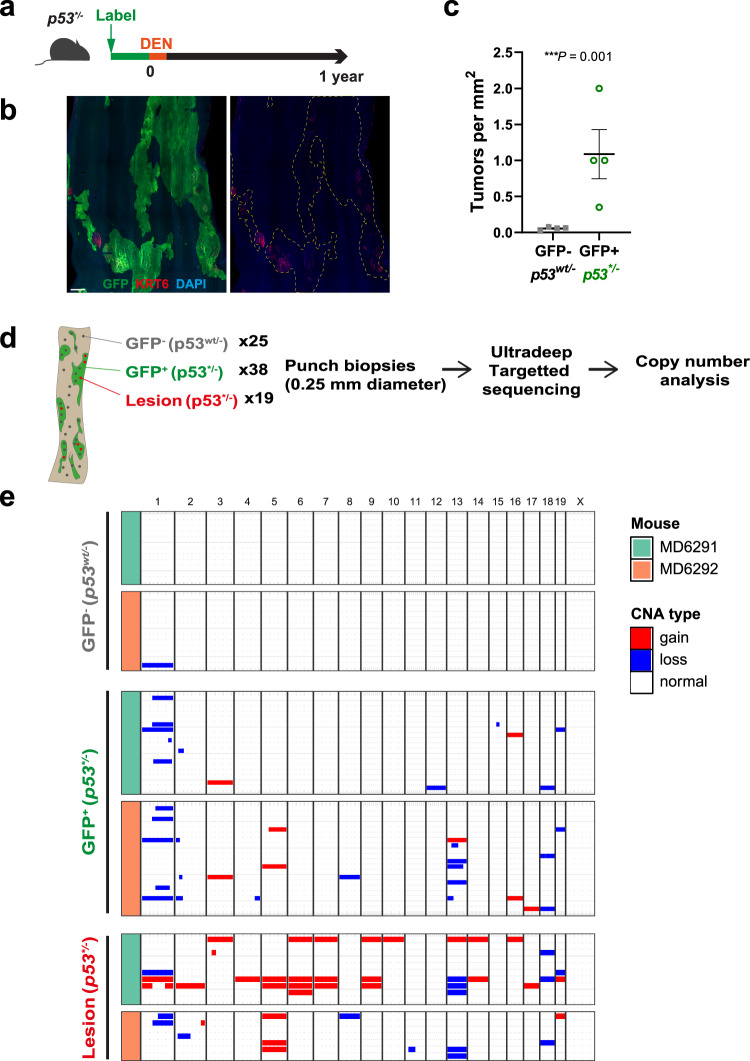
Effect of mutagenesis and aging p53^*/−^ epithelium. **a** Protocol: Induced p53^*/−^ mice were treated with DEN for 2 weeks and aged. **b** Confocal image of p53^*/−^ induced epithelium at 1 year post DEN treatment. Typical example from *n* = 4 mice. Green, GFP; red, KRT6; blue, DAPI. Scale bar, 400 µm. **c** Number of tumours found in p53^*/−^ (GFP+) and *p53*^*wt/−*^ (GFP−) area. Error bars are mean ± s.e.m. *n* = 4 mice, *p* value was determined by two-tailed ratio paired t-test. **d** Sequencing analysis of DEN treated p53^*/−^ epithelium at 1 year time point. Micro-punch biopsies were taken from physiologically normal epithelium, GFP-negative p53^*/−^ or GFP-positive p53^*/−^ clone area, and from lesions (p53^*/−^). Chromosomal alterations were analyzed using targeted sequencing data. **e** Summary of copy number alterations. Gain and loss of chromosomes was predominantly detected in p53^*/−^ clone area and lesions which arose from p53^*/−^ cells. *n* = 2 mice; 25 biopsies for *p53*^*wt/−*^, 38 biopsies for p53^*/−^ clone areas, 19 biopsies for lesions. See Supplementary Data 25 and 26.

Since the genotype of human oesophageal SCC suggests they arise from *TP53* mutant cells with LOH, we evaluated the effect of mutagenesis and aging of p53^*/−^ epithelium. *Ahcre*^*ERT*^p53^*/−^ mice were induced to generate p53^*/−^ clones. Animals were exposed to a lower dose of DEN than used above, to reduce the risk of malignancy in p53^*/−^ cells recombined outside the oesophagus, and then aged (Fig. 6a). At 1 year, the oesophagus was mosaic, with unrecombined *p53*^*wt/−*^ areas and p53^*/−^ epithelium allowing a direct comparison of tumour burden in the same animal. This showed the number of tumours was significantly higher in GFP+ p53^*/−^ area than *p53*^*wt/−*^ area (Fig. 6b, c). Furthermore, when tumours and adjacent epithelium were sequenced to detect CNA, there was minimal CNA in *p53*^*wt/−*^ epithelium, but extensive CNA in both p53^*/−^ epithelium and the tumours within it (Fig. 6d, e).

Taken together, above results show that the heterozygous p53* mutant promotes tumour growth, with a modest effect on CNA, whereas p53* combined with loss of the second *p53* allele in the setting of low dose mutagen exposure increases the number of tumours and results in marked chromosomal instability both in normal epithelia and in tumours.

## Discussion

Our results establish that the p53* mutation has discrete roles at distinct stages of carcinogenesis. Heterozygous p53* progenitors acquire a proliferative advantage through a bias in cell fate, so that on average mutant cell divisions produce an excess of progenitors over differentiated cells, a mechanism shared with p53* clonal expansion in the epidermis^19^. This fate imbalance is sufficient to drive clonal expansion in both wild type and mutagenized epithelium which contains competing clones of varying fitness^30^. The molecular basis by which p53* alters progenitor cell fate is not known, but p53 interacts with multiple known regulators of keratinocyte differentiation, such as p63, which in turn induces *Klf4*, a transcription factor required for oesophageal differentiation^34,35^.

Biasing progenitor cell fate in favour of proliferation is a cellular mechanism shared by diverse mutations that cause oesophageal clonal expansion, including activating *Pik3ca*^*H1047R*^, *Notch1*^*wt/−*^ and dominant negative mutant *Maml1*^13,24,25^. In common with these other mutants, after an initial phase of expansion p53^*/wt^ has a minimal epithelial phenotype. Once mutant cells are surrounded by cells that are themselves mutant, cell fate bias decreases and reverts towards balance allowing the epithelium to retain tissue integrity^30^. Furthermore, we observed no increase in age-associated mutation rate above that of wild type oesophagus or any detectable CNA in p53^*/wt^ epithelium. Heterozygous p53* induced at clonal density thus expands to establish a mutant population that persists long term within the normal appearing oesophagus, paralleling the behaviour of this mutant in the epidermis^19^.

Clonal competition plays a key role in restricting the expansion of the *p53* mutant population. Comparison of growth of p53^*/wt^ cells in a wild type and mutagenized background shows that the size of p53^*/wt^ clones is substantially reduced by the presence of competitive mutants such as *Notch1* in adjacent epithelium, resulting in a mutational landscape similar to that of ageing humans^2,25,30,33^. Overall, our findings explain the slow accumulation of heterozygous *TP53* mutants over decades in the dense mutational patchwork that is ageing normal human oesophageal epithelium.

90% of oesophageal dysplastic lesions, as well as cancers, exhibit disruption of both *TP53* alleles arguing lesions emerge from clones that lose the wild type *TP53* allele within the *TP53* heterozygous mutant population^10,11,16^. Clones with *TP53* LOH are rare in normal human oesophagus and the absence of the CIN and aneuploid giant cell phenotype in the ageing oesophagus of highly induced p53^*/wt^ mice argues that spontaneous biallelic loss with resulting in clonal expansion is infrequent in mice. This contrasts with *Notch1*, where large clones carrying mutations in the second allele of *Notch1* are frequent in aging heterozygous *Notch1* mouse oesophagus, reflecting an increase in fitness of that occurs upon loss of the second allele. In humans most *NOTCH1* clones in normal epithelium have LOH of *NOTCH1* and spread to occupy the majority of the normal epithelium by middle age^25^. If *TP53* mutants gained fitness upon loss of the second allele in a similar manner to mutant *NOTCH1* clones, the proportion of epithelium qualified for tumour progression would be much higher than is observed.

Once an area of *p53* mutant epithelium with LOH does develop, genome instability in the form of giant polyaneuploid like cells and clonal expansions carrying CNA occurs. Transcriptomic analysis and centrosomal imaging point to disruption of cytokinesis underlying the development of aneuploidy cells in cells lacking wild type *p53*, consistent with findings in *p53*^*−/−*^ epithelial organoid cultures^36^. Giant polyaneuploid cells have been proposed as a population that resists cancer treatment and can regenerate tumours, but their significance in oesophageal pre-cancer is unclear^27^. The scarcity of *TP53* mutant clones with LOH in humans makes it difficult to know if such a phenotype occurs prior to transformation in human oesophagus, but marked CNA is an early feature of both premalignant lesions and SCC with biallelic *TP53* loss^10^.

The suggestion that p53^*/−^ cells are better qualified for transformation than p53^*/wt^ seems borne out in the mutagenesis studies performed here. Tumour size was increased in p53^*/wt^ mutant epithelium, arguing the mutation promotes the growth of tumours after their initiation by other mutations, but there was no increase in the number of tumours and minimal CNA. By contrast, after mutagen treatment there was extensive CNA in both p53^*/−^ epithelium and the tumours derived from it compared with p53^*/wt^ tissue, and the p53^*/−^ tumours were more numerous.

**Fig. 7:**
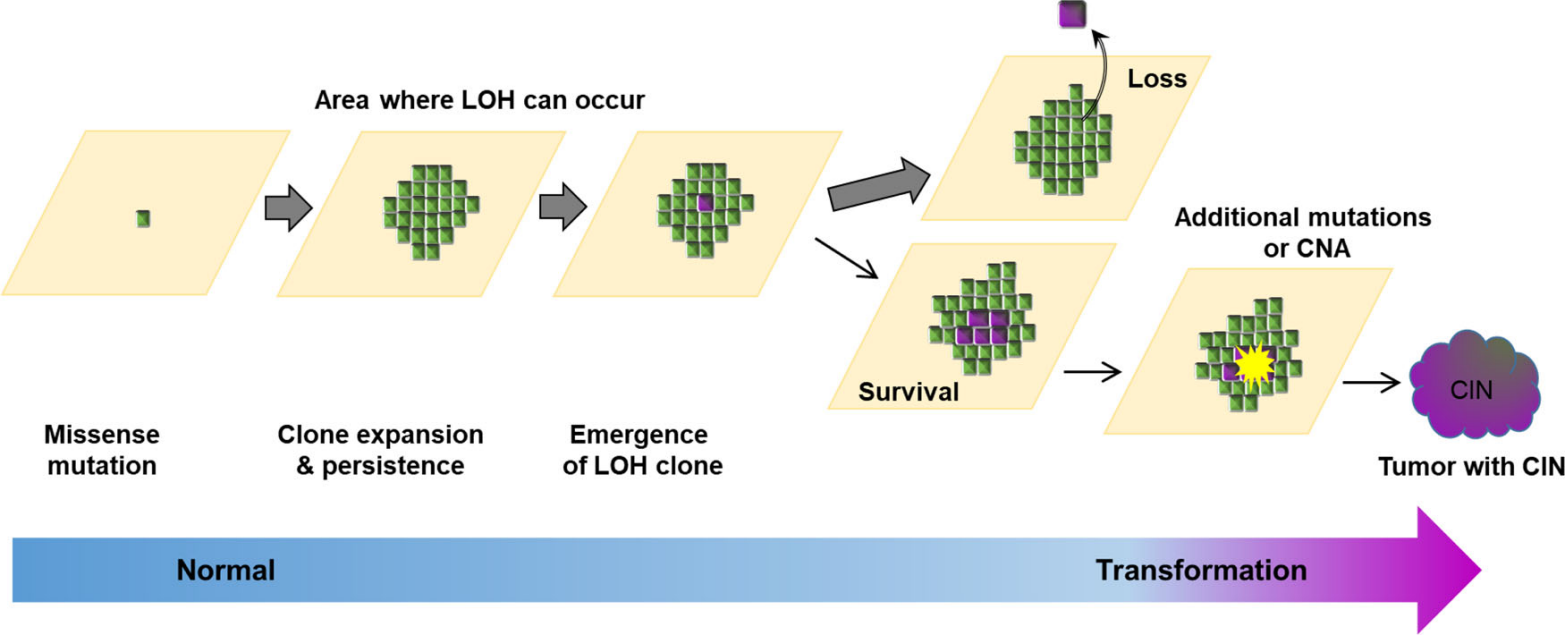
Mutant *p53* in normal epithelia in carcinogenesis. Heterozygous *p53* mutation in single cells in normal epithelia confers a proliferative advantage, clonal expansion, and a population of mutant cells that persists to undergo LOH. Clones with LOH are rare, but those that persist may acquire CNA and progress to form tumours. Once a tumour has formed, *p53* mutation enhances tumour growth.

In conclusion, these findings highlight how oncogenic *p53* mutations have distinct effects at different stages of transformation (Fig. 7). The promotion of clonal expansion results in minimal disruption to normal epithelium, indeed is tolerated in humans over decades. Heterozygous mutants have a low risk of transformation and do not contribute to genome instability. Loss of the second allele does not appear to initiate tumour formation but allows additional genome alterations that may promote clonal expansion and/or tumour formation^37–39^. Within established tumours, *p53* mutation is advantageous, favoring tumour growth. The multiple facets of *p53* mutant biology must be placed in the context of clonal competition which limits the expansion of the *p53* mutant population in order to understand epithelial carcinogenesis.

## Methods

### Animals

Experiments were conducted according to UK government Home Office project licences PPL22/2282, PPL70/7543, and PPL4639B40. Animals were housed in individually ventilated cages and fed on standard chow and maintained on a *C57/Bl6* genetic background, at specific and opportunistic pathogen-free health status, and were immune competent. Adult mice were used for in vivo experiments and no animals were involved in previous experiments and were drug naive prior to the start of experiments. Both male and female animals were used for experiments.

*Ahcre*^*ERT*^*Rosa*^*flEYFP/wt*^*, Ahcre*^*ERT*^*Rosa26*^*flconfetti/wt*^*, and Ahcre*^*ERT*^*Trp53*^*flR245W-GFP/wt*^ knock-in mice were generated as previously described. *Ahcre*^*ERT*^*Trp53*^*flR245W-GFP/−*^ mice were generated by crossing *Ahcre*^*ERT*^*Trp53*^*flR245W-GFP/flR245W-GFP*^ with heterozygous *p53* knockout mice, *p53*^*+/−*^.

In the *Ah*^*creERT*^ line, transcription from a transgenic *CYP1A1* (arylhydrocarbon receptor, *Ah*) promoter is normally tightly repressed (Kemp et al., 2004). Following treatment with the non-genotoxic xenobiotic β-napthoflavone the *Ah* promoter is induced and a *cre* recombinase-mutant oestrogen receptor fusion protein (creERT) is expressed. In the presence of tamoxifen, the creERT protein enters the nucleus to mediate recombination.

For lineage tracing experiments, the relevant floxed reporter line which was crossed onto the *Ah*^*creERT*^ strain were induced by a single interaperitoneal (i.p) injection of 80 mg/kg β-napthoflavone (βNF) and 1 mg tamoxifen (Tam) at 11–16 weeks of age. For some experiments, in order to achieve optimal clone density, xx mg/kg βNF and xx mg Tam was used for *Ahcre*^*ERT*^*Trp53*^*flR245W-GFP/wt*^ and *Ahcre*^*ERT*^*Trp53*^*flR245W-GFP/−*^ GFP and YFP expressing clones were visualized by immunostaining with an anti-GFP antibody.

### Mutagen treatment

To induce mutations in the oesophageal epithelium, mice were administered with DEN in sweetened drinking water (40 mg/L) for 3 days a week (Monday, Wednesday, and Friday) for 8 weeks. Control mice received sweetened water as vehicle for the length of the treatment.

### Whole mount sample preparation

Mouse oesophagus was dissected, cut longitudinally and the muscle layer removed by pulling with forceps. Entire tissue was incubated for 2.5 h in 5 mM EDTA at 37 °C before and then the epithelium was carefully separated from underlying submucosa using fine forceps followed by fixation in 4% paraformaldehyde for 30 min at room temperature. The tissues were then washed in PBS and stored at 4 °C.

### Immunofluorescence

For staining, wholemounts were blocked in staining buffer (0.5% Bovine Serum Albumin, 0.25% Fish Skin Gelatin, and 0.5% Triton X-100 in PBS with 10% goat or donkey serum according to the secondary antibody used) for 1 h at room temperature. Samples were incubated with primary antibody in staining buffer overnight, washed in PBS containing 0.2% Tween-20 four times, incubated with fluorchrome-conjugated secondary antibody for 2 h at room temperature and washed as before After the final wash, samples were incubated with 40,6-diamidino-2-phenylindole (DAPI, 1 µg ml^−1^) or SiR-Hoechst (SiR-DNA) (Spirochrome, SC007) to stain cell nuclei and mounted on slides using Vectashield Mounting Medium (Vector Labs).

EdU incorporation was detected with a Click-iT imaging kit (Life Technologies) according to the manufacturer’s instructions.

### Antibodies

Primary antibodies used in this study are as follows: anti-GFP/YFP (ThermoFisher Scientific, A10262) 1:1000, anti-Cytokeratin 14 (Covance, PRB-155P) 1:2000, anti-Cytokeratin 6 (Biolegend, 905701) 1:2000, anti-pericentrin (abcam, ab4448) 1:1000, anti-CD45 (Biolegend, 103102) 1:500, anti-CD31 (abcam, ab7388) 1:1000, anti-Loricrin (Covance PRB-145P) 1:2000, Alexa Fluor 647 anti-CD49f (Biolegend, 313610) 1:250.

All secondary antibodies are Alexa Fluor conjugated and diluted 1:1000 in staining buffer: 488 anti-chicken (Jackson ImmunoResearch, 703-545-155), 555 anti-rabbit (ThermoFisher Scientific, A31572), 647 anti-mouse (ThermoFisher Scientific, A32787), 488 and 647 anti-rat (ThermoFisher Scientific, A21208 and A48272).

### Frozen sections and histology

Tissues were frozen in O.C.T and cut at 14 µm thickness in the cryostat. Sections were then fixed in 4% paraformaldehyde in PBS for 10 min and washed in PBS before staining with hematoxylin and eosin or immunostaining as described above.

### Pathology of macroscopic tumours

Pathological assessment of tumours was carried out by MRC Metabolic Diseases Unit [MC_UU_00014/5].

### Imaging

Confocal images were acquired on Leica TCS SP5 II or SP8 microscopes using 10×, 20×, 40×, or 64× objectives. Typical settings for acquisition of z stacks were optimal pinhole, line average 4 scan speed 400 Hz and a resolution of 1024 × 1024 pixels or 2048 × 2048 pixels. Image analysis was performed using Volocity 6 or 6.3 image processing software (Perkin Elmer).

### Clonal lineage tracing

After immunostaining wholemounts, clones were imaged by confocal microscopy and the number of basal and suprabasal cells in each clone counted in live acquisition mode.

### Primary keratinocyte 3D culture

Mouse oesophagus with the muscle removed were cut into small pieces (2 mm^2^) and placed on a transparent ThinCert^TM^ insert (Grenier Bio-One) with the epitheliem facing upward and the submucosa stretched over the membrane. Inserts in the culture plates were then placed at 37 °C for at least 30 min to ensure the attachment of oesophagus explants to the membrane followed by culture in complete FAD medium (50:50) 4.5 g/L D-Glucose, Pyruvate, L-Glutamine D MEM (Thermo Fisher Scientific, 11995-065): D-MEM/F12 (Thermo Fisher Scientific, 31330-032), supplemented with 5 µg/ml insulin (Merck, I5500), 1.8 × 10^−4^ M adenine (Merck, A3159), 0.5 µg/ml hydrocortisone (Merck, 386698), 1 × 10^−10^ M Cholera toxin (Merck, C8052), 10 ng/ml Epidermal Growth Factor (EGF, PeproTech EC Ltd, 100-15), 5% feral calf serum (PAA Laboratories, A15-041), 5% Penicillin-Streptomycin (Merck, P0781), 1% Amphotericin B (Merck, A2942) and 5 µg/ml Apo-Transferrine (Merck, T2036). Explants were removed after 7 days once half of the membrane had been covered with keratinocytes and the culture was maintained by changing media every three days. When culture established the confluent monolayer, Cholera toxin, EGF and hydrocortisone was removed from the medium to promote differentiation and stratification resulting in multi-layered keratinocyte sheet.

### Virus transduction

To induce cre-recombination in vitro, cultured keratinocytes were infected with Adenovirus encoding Cre recombinase (Ad-CMV-iCre, Vectorbiolabs, #1045 UK). Briefly confluent keratinocyte culture was dissociated by trypsinization, resuspended in adenovirus-containing medium supplemented with polybrene, and seeded in the insert. Following 24 h incubation at 37 °C, cells were washed and then cultured in complete FAD medium until confluent. For the experiments, cells were maintained in FAD media without cholera toxin, EGF and hydrocortisone. For non-recombined control, similarly cells were infected with empty Adenovirus.

### In vitro cell competition assay

Oesophageal keratinocytes were isolated from *Trp53*^*flR245W-GFP/wt*^ and *Trp53*^*flR245W-GFP/−*^ mice and cultured in 3D and p53* mutation was induced fully by adenovirus infection as described above. As *p53*^*wt/wt*^ and *p53*^*wt/-*^, same set of cells infected with empty adenovirus were used. Established cultures were trypsinized, mixed 1:3 (p53^*/wt^ or p53^*/−^: *p53*^*wt/wt*^ or *p53*^*wt/−*^) and seeded in complete FAD medium. When cultures are fully confluent, cholera toxin, EGF and hydrocortisone was removed from the medium (day 0) and maintained for 28 days. Proportion of GFP+ p53^*/wt^ or p53^*/−^ cells in culture was determined by flow cytometry at day 0 and day 28, and data were presented as fold change. Duplicate cultures were immunostained with anti-GFP as described above and imaged by confocal microscopy.

### Flow cytometry

Flow cytometry was carried out using CytoFLEX (Beckman Coulter Life Sciences) flow cytometer and data was acquired by CytoExpert software (Beckman Coulter Life Sciences). GFP fluorescence was collected using the 488 nm laser and a 525/40 bandpass filter. Data was analyzed using FlowJo v10.6.1 (Benton Dickinson). Gating strategy is shown in Supplementary Fig. 8: FSC/SSC gating was applied to identify cell population of interest and to exclude debris. Doublet and multiplet cells were excluded by FSC-A/FSC-Width gating. Boundaries of GFP positive and negative population are defined using control samples.

### RNA-Seq and expression analysis

Total RNA was extracted from 3D cultures of mouse primary keratinocytes using RNeasy Micro Kit (QIAGEN, UK), following the manufacturer’s instructions, including DNase digestion. For RNAseq, libraries were prepared in an automated fashion using an Agilent Bravo robot with a KAPA Standard mRNA-Seq Kit (KAPA BIOSYSTEMS). In house adaptors were ligated to 100–300 bp fragments of dsDNA. All the samples were then subject to 10 PCR cycles using the sanger_168 tag set of primers and paired-end sequencing was performed on Illumina’s HiSeq 2500 with 75 bp read length.

Reads were aligned using STAR (v2.5.3a) to the GRCm38 mouse genome (GCA_000001635.8, GCF_000001635.26, [https://www.ncbi.nlm.nih.gov/data-hub/genome/GCF_000001635.26/] Alignment files were sorted and duplicate-marked using Biobambam2 (v2.0.54). Read counting was performed using the script htseq-count (v0.6.1p1)^40,41^ and differential gene expression was analyzed using DESeq2 (v1.2.0)^42^. Downstream analysis and visualization was performed using R (v3.5.1, https://www.R-project.org/) with the packages pheatmap (v1.0.10, https://CRAN.R-project.org/package=pheatmap), clusterProfiler (v3.8.1, https://www.bioconductor.org/packages/devel/bioc/html/clusterProfiler.html)^43^ with mouse annotation provided by org.Mm.eg.db (v3.6.0, http://bioconductor.org/packages/org.Mm.eg.db/). Heatmaps were generated from the Transcript Per Million (TPM) values using Morpheus (https://software.broadinstitute.org/morpheus/).

### DNA *sequencing*

#### 2 mm^2^ gridded samples

Oesophageal epithelium prepared as described above was cut into a contiguous array of 2 mm^2^ biopsies. DNA was extracted from samples using QIAMP DNA micro kit (Qiagen, catalogue no. 56304) following the manufacturer’s instructions. DNA from the ears of the same mice was also extracted in the same way and used as germline controls.

#### Low input sequencing samples

Oesophageal epithelium was stained for GFP to identify *p53* mutant areas and 0.25 mm diameter samples were taken from GFP positive, GFP negative and microscopic lesions using a brain punch biopsy (Stoelting Europe) under fluorescent dissection microscope. Frozen sections of macroscopic tumours were stained with H&E and area of interest was scraped off from the slide using scalpel. Samples were extracted using the Arcturus Picopure Kit (Applied Biosystems) following the manufacturer’s instructions.

### Sequencing and alignment

Targeted-enriched samples were multiplexed and sequenced on the Illumina HiSeq 2000 v4 platform using paired-end 75-base pair reads. WGS was also performed on HiSeq 2000 v4 platform using paired-end 125-base pair reads. Paired-end reads were aligned to the GRCm38 reference with BWA-MEM (v.0.7.16a & v0.7.17, https://github.com/lh3/bwa). Samples were collected and sequenced over 2 years during which BWA-MEM was updated from v0.7.16a to v0.7.17. As noted in the release notes for v0.7.17 this release adds features but did not alter the alignment output if parameters were not changed, hence remapping is not required for the re-run v0.7.16a data sets. Optical and PCR duplicates were marked using Biobambam2 (v.2.0.86, https://gitlab.com/german.tischler/biobambam2). Samples were mapped to the GRCm38 reference. Mean depth of coverage for each set of sequencing can be found in Supplemental data 12, 20, 22, and 24.

### Custom baitset design

A custom bait capture to target the exonic sequences of 142 genes was designed using Agilent SureDesign. The list of genes selected for ultra-deep targeted sequencing is shown below:

*abcb11, abcc2, adam10, aff3, ajuba, akt1, apob, arid1a, arid2, arid5b, asxl1, atm, atp2a2, atrx, b2m, bcl11b, braf, brca2, cacna1d, card11, casp8, ccnd1, cdh1, cdkn2a, cobll1, col12a1, crebbp, csmd2, ctcf, ctnnb1, ctnnd1, cul3, cyld, dclk1, dclre1a, ddr2, dicer1, dll1, dll3, dnm2, dnmt3a, dst, dtx1, dtx3, egfr, eif2d, ep300, epha2, erbb2, erbb3, erbb4, ezh2, fat1, fat2, fat3, fat4, fbxo21, fbxw7, fgfr1, fgfr2, fgfr3, flt3, grin2a, hmcn1, hras, huwe1, hydin, itch, jag1, jag2, kdm6a, kdr, keap1, kit, klrc3, kmt2c, kmt2d, kras, krtap4-9, lfng, lrp1b, lrp2, maml1, maml2, maml3, met, mfng, mib1, mtor, nf1, nf2, nfe2l2, notch1, notch2, notch3, notch4, nras, nsd1, numb, numbl, pik3ca, prex2, psen1, psen2, psme4, ptch1, pten, rac1, rasa1, rb1, rbpj, rfng, rhbdf2, rita1, robo1, robo2, ros1, rpgrip1, rpl10, ryr2, scn10a, setd2, slit2, smad4, smarca4, smo, sox2, spen, stat5b, sufu, tert, tet2, tgfbr1, tgfbr2, trp53, trp63, tsc1, vhl, vmp1, zan, zfhx3, zfp750*.

### Variant calling

#### ShearwaterML

Somatic mutations are generally identified by detecting mismatches between samples (typically comparing tumour with normal samples sequenced at relatively low coverage of 20–40×). Here, in order to identify somatic mutations present in a small fraction of cells within the samples we used the ShearwaterML algorithm from the deepSNV package (v1.21.3, https://github.com/gerstung-lab/deepSNV) to call for mutation events on ultra-deep targeted data^30,33,44^. Instead of using a single-matched normal sample, the ShearwaterML algorithm uses a collection of deeply-sequenced normal samples as a reference for variant calling that enables the identification of mutations at very low allele frequencies. For the normal panel, we used 28 germline samples from ears of the mice analyzed in this study and additional mice. Summed mean depth of coverage was 6848× and 4394× for 2 mm^2^ gridded samples and 0.25 mm diameter punch samples, respectively.

#### CaVEMan and Pindel

For sequencing data of 0.25 mm diameter samples, variants were called using the CaVEMan (v1.13.14 & v1.14.0) and Pindel (v3.3.0) algorithms^45,46^. For SNVs, CaVEMan was run with the major copy number set to 10 and the minor copy number set to 2. Only SNVs that passed all CaVEMan filters were retained. CaVEMan v1.14.0 was released mid-project and was utilized as it offered additional error checking and speed improvements without altering the results from v1.13.14. Additional filtering to remove mapping artefacts associated with BWA-MEM were: the median alignment score of reads supporting a variant had to be at least 140 and the number of clipped reads equal to zero. In addition, the proportion of mutant reads present in the matched sample also had to be zero. Variants with at least one mutant read present in the matched sample were also removed. Two SNVs called at adjacent positions within the same sample were merged to form a doublet-base substitution if at least 90% of the mapped DNA reads containing at least one of the SNV pair contained both SNVs. Small (<200 bp) insertions and deletions were called using Pindel. Only indels that passed all Pindel filters were kept.

Variants were annotated using VAGrENT (v3.7.0)^47^. Full lists of called and pass-filter variants are in Supplementary Data 14, 22, 24, and 26.

#### Merging of mutant clones from contiguous samples

In order to avoid counting the same mutation multiple times and to obtain a more accurate estimate of clone sizes, clonal mutations that spanned between two or more adjacent biopsies were merged and considered as singular events. To do this we calculated the mean number of shared mutations between biopsies at increasing distances, since the immediately adjacent samples are predicted to have more shared mutations than distant samples. We decided to merge mutations common between samples closer than 3 mm, as the number of shared mutations plateau at distances greater than this.

#### Mutation burden

The average number of mutations per cell in a given sample was estimated from the VAFs and the number of bases within the bait set with synonymous mutations (dubbed as synonymous footprint), as described previously^30^. When using targeted sequenced data, the mutation burden can be inflated by the alterations in strongly selected genes. Therefore, only the synonymous mutations were used for this calculation.

#### Clone sizes and coverage

The size of mutant clones within each sample can be calculated from the area of the biopsy (2 mm^2^) and the fraction of cells carrying a mutation within the sample, as described previously^30,33^. The lower (=VAF) and upper (=2xVAF) bound estimates of the percentage of epithelium covered by clones carrying non-synonymous mutations in a given gene was calculated for each biopsy, this range allows for uncertainty in copy number. The fraction of epithelium covered by the mutant genes was then calculated from the mean of summed VAF (capped at 1.0) of all the biopsies in the same tissue.

### Gene selection (d*N*/d*S*)

We used the maximum-likelihood implementation of the dNdScv algorithm (v0.0.1.0, https://github.com/im3sanger/dndscv) to identify genes under positive selection^30^. dNdScv estimates the ratio of non-synonymous to synonymous mutations across genes, controlling for the sequence composition of the gene and the mutational signatures, using trinucleotide context-dependent substitution matrices to avoid common mutation biases affecting dN/dS. Values of d*N*/d*S* significantly higher than 1 indicate an excess of nonsynonymous mutations in that particular gene and therefore imply positive selection, whereas dN/dS values significantly lower than 1 suggest negative selection. In our experimental set up, only mutations that reach a minimum size are detected by deepSNV. Therefore, a significant value of d*N*/d*S* > 1 indicates that a clone that acquires a non-synonymous mutation in that particular gene will have a higher probability to reach a detectable clone size as compared to a synonymous mutation in the same gene. Hence, genes with dN/dS > 1 are considered drivers of clonal expansion.

### Mutational spectra and signature

Mutational spectra for single base substitutions were plotted and compared to 65 known mutational signatures using linear decomposition with the deconstructSigs R package (v1.9.0, https://github.com/raerose01/deconstructSigs)^48^. The mutational spectra of DEN-treated samples were highly consistent, precluding deconvolution into separate signatures (either known or de novo). Transcriptional strand bias was analyzed using MutationalPatterns (v3.4.0, https://bioconductor.org/packages/release/bioc/html/MutationalPatterns.html)^49^.

### Copy number analysis

The copy number analysis workflow was conceptually very similar to that used in^30^. That paper described analysis of whole genome amplified exome sequenced triples. Here we utilize whole genome and targeted sequencing, which required adaptation of the workflow. For clarity and completeness, the workflow is described in full here.

#### Whole genome sequencing

A modified version of QDNAseq (https://github.com/ccagc/QDNAseq/) was used to call changes in total copy number from the low coverage (mean coverage 1.8×) whole genome sequencing data^50^. QDNAseq was modified to include the correction of the coverage profile of the sample of interest by that of a matched control. The procedure to call gains and losses is as follows: First sequencing reads were counted per 100 kb bins for both the sample of interest and the matched control. The bin-counts were then combined into coverage log ratio values to obtain what is commonly referred to as “logR”. The calculation of logr is implemented similarly to how the Battenberg copy number caller calculates these values^51^: first the bin-counts from the sample of interest were divided by the control bin-counts to obtain the coverage ratio; the coverage ratio was then divided by the mean coverage ratio and finally the log2 was taken to obtain logr. The regular QDNAseq pipeline is then applied: First a correction of the logr for GC content correlated wave artifacts, then segmentation and finally calling of gains and losses.

#### Targeted sequencing

The workflow to analyze targeted sequencing data differs slightly from the above whole genome workflow. When performing targeted sequencing, only a subset of the genome is actively targeted and yields high coverage. The remainder of the genome is typically covered by much shallower, off-target coverage^52^. Removal of the targeted regions (typically a small proportion of the genome) effectively yields a shallow whole genome sequencing sample.

The read counting step was therefore adapted to only count off-target reads. We first counted all reads per 1Kb bins along the genome, then removed any bin that overlaps with the start/end coordinates of a region contained within the kit and mapped the remaining onto bins of 1 Mb. This procedure was applied to both the sample of interest and the matched control, after which the construction of logr normalizes out the fact that some bins span slightly less than 1 Mb due to removal of the targeted regions. The above-described pipeline is subsequently run to obtain copy number calls.

A comparison of calls between samples from whole genome and targeted sequencing from the same mice shows good correspondence of the overall picture gained from copy number analysis (Fig. 4h and Supplementary Fig. 5f).

#### Post-hoc filtering of calls

A post hoc filtering step was subsequently applied to obtain robust copy number calls. The calls from both whole genome and targeted sequencing were required to constitute an alteration in at least 10% of sequenced cells and be at least 20 Mb in size.

### TCGA analysis

The TCGA ESCA project dataset contains the largest publicly available complete dataset with SNV, CNV, and LOH calls from whole genome sequencing of ESCC samples^53,54^. Sample metadata was obtained from the NIH GDC data portal (https://portal.gdc.cancer.gov/). Within the ESCA project filters were applied to select only SCC samples with a matched blood normal sample. Application of filters resulted in 88 samples remaining for analysis. Somatic SNV and CNV calls were also obtained from the GDC portal. LOH calls for TCGA were published previously and are also available from the GDC pancan portal (https://gdc.cancer.gov/about-data/publications/pancan-aneuploidy)^53^. Median age across the ESCC sample cohort was 57, atypical of the incidence in Europe which peaks in the age group 85 to 89 for females and above 90 for males (https://www.cancerresearchuk.org/health-professional/cancer-statistics/statistics-by-cancer-type/oesophageal-cancer/incidence.)

Variants overlapping *TP53* were extracted for each of the samples and were summarized in Supplementary Data 1. For each sample the most severe SNV consequence in *TP53* was identified in the order: Dominant negative, nonsense, missense, other & none. Identified dominant negative mutations defined as the following previously identified in literature: p.R175H, p.Y220C, p.M237L, p.R248Q, p.R248W, p.R273H, p.R282W & p.R249S^55,56^.

### Statistical analysis

Data are expressed as mean values ± s.e.m. *p* < 0.05 was considered statistically significant.

Statistical analysis was performed using the Graphpad Prism software and can be found in Supplementary Data. No statistical method was used to predetermine sample size. The experiments were not randomized. The investigators were not blinded to allocation during experiments or outcome assessment.

### Spatial simulations of p53^*/wt^ mutant clone growth in mouse oesophagus

p53^*/wt^ mutant cell behaviour was modelled as a non-neutral single progenitor (SP) model with a fate imbalance (*δ*) in the mutant population^14^. This imbalance leads to an increased probability of symmetric divisions producing two mutant progenitors, effectively providing a fitness advantage over wild-type cells.

Here we adapted the spatial single progenitor model described in Kostiou et al. implemented in NetLogo^57,58^. Briefly, we simulate the imbalanced single progenitor as a two-dimensional stochastic cellular automaton (CA) model in a hexagonal lattice (Supplementary Fig. 2a). Basal cells were simulated on a lattice of 10,000 cells (1% of the area of adult mouse oesophagus), with periodic boundary conditions. Each simulation was repeated 100 times. To take account of local crowding events lattice site may be occupied by either by no cell, single cells, or pairs of cells. Doubly occupied sites transition to single occupancy once immediately adjacent empty sites become available or through an instant “extrusion” event, if one cell is committed to the differentiated fate. Based on previous observations of p53^*/wt^ mutant cell growth in mouse skin, mutant cells were set to respond to adjacent double-occupancy sites by reducing their propensity to symmetric division giving dividing cells (Figs. 1b, 2f)^58^.

#### Model parameter estimation

To estimate the key parameter representing fate imbalance, following experimental observations that *p*53^*/wt^ cells divide and stratify at the same rates as their wild type counterparts, we made the simplifying assumption that other parameters remain unchanged in the mutant population. This can be estimated from a linear regression applied to the *ln* (*average clone size*)^59^, where the slope of the line is a function of the known parameters and the imbalance.

We used parameter values describing the homoeostatic cell population dynamics in mouse oesophageal epithelium provided by Doupe et al.^14^. To test the robustness of these parameters, two more parameter combinations are were taken from different experiments the mouse oesophagus^15^. All available parameter sets proposed for this tissue were simulated (Supplementary Fig. 2). The parameter combinations and resultant imbalance (δ) are shown in Table 1:

**Table 1:** Estimated parameter values

$r$	$\varGamma$	$\lambda$	$\delta$	Reference
0.1	3.5/week	1.9/week	0.29	^15^
0.06	3.7/week	2.9/week	0.32	^16^
0.1	5.4/week	2.9/week	0.19	^16^

We further tested the robustness of the parameter estimates by perturbing estimates of *λ* and *r*, find that there was a broad agreement between simulations and observations (Supplementary Fig. 2b, c).

Analysis of the simulated *p53* mutant cell populations in the basal layer of mouse oesophagus demonstrates that the model is in quantitative agreement with the experimental values and is broadly robust to the different parameters proposed for this tissue (Supplementary Fig. 2). This confirms that a spatial non-neutral single progenitor model with cell density-dependent feedbacks can recapitulate the observed *p53* mutant behaviour in the mouse oesophageal epithelium.

### Reporting summary

Further information on research design is available in the Nature Research Reporting Summary linked to this article.

## Supplementary information

Supplementary Information

Peer Review File

Reporting Summary

Description of Additional Supplementary Files

Supplementary data 1-26

## Data Availability

The sequencing data sets in this study are publicly available at the European Nucleotide archive (ENA) Accession numbers for RNAseq data on https://www.ebi.ac.uk/ena are as follows: p53^wt/wt^, ERS1432686, ERS1432689, ERS1432688, ERS1432703, ERS1432704, ERS1432705; *p53*^**/wt*^, ERS1432636, ERS1432637, ERS1432634, ERS1432638, ERS1432635, ERS1432639; *p53*^*wt/−−*^, ERS1432650, ERS1432651, ERS1432656, ERS1432654, ERS1432657, ERS1432658; *p53*^**/−*^, ERS1432672, ERS1432670, ERS1432671, ERS1432673, ERS1432674, ERS1432676. Accession numbers for targeted DNA sequencing samples and Whole genome sequencing are ERP129331 and ERP129332 respectively. All source data for Figures is provided in Supplementary Data files. All analysed sequencing data is provided in Supplementary Data files.

## Code availability

The pipeline code and modified QDNAseq package are available at https://github.com/sdentro/qdnaseq_pipeline and https://github.com/sdentro/QDNAseq/tree/dev respectively.
