# Supplementary material for: p53 mutation in normal esophagus promotes multiple stages of carcinogenesis but is constrained by clonal competition
Source: Nat Commun. 2022 Oct 20;13:6206. doi: 10.1038/s41467-022-33945-y (PMC9584949; doi:10.1038/s41467-022-33945-y)
Supplement: Supplementary file 1 — Supplementary Information [file 41467_2022_33945_MOESM1_ESM.pdf]

p53 mutation in normal esophagus promotes multiple stages of carcinogenesis but is constrained by clonal competition

Kasumi Murai, Stefan Dentro, Swee Hoe Ong, Roshan Sood, David Fernandez-Antoran, Albert Herms, Vasiliki Kostiou, Irina Abnizova, Benjamin A Hall, Moritz Gerstung, Philip H Jones

Supplementary Information

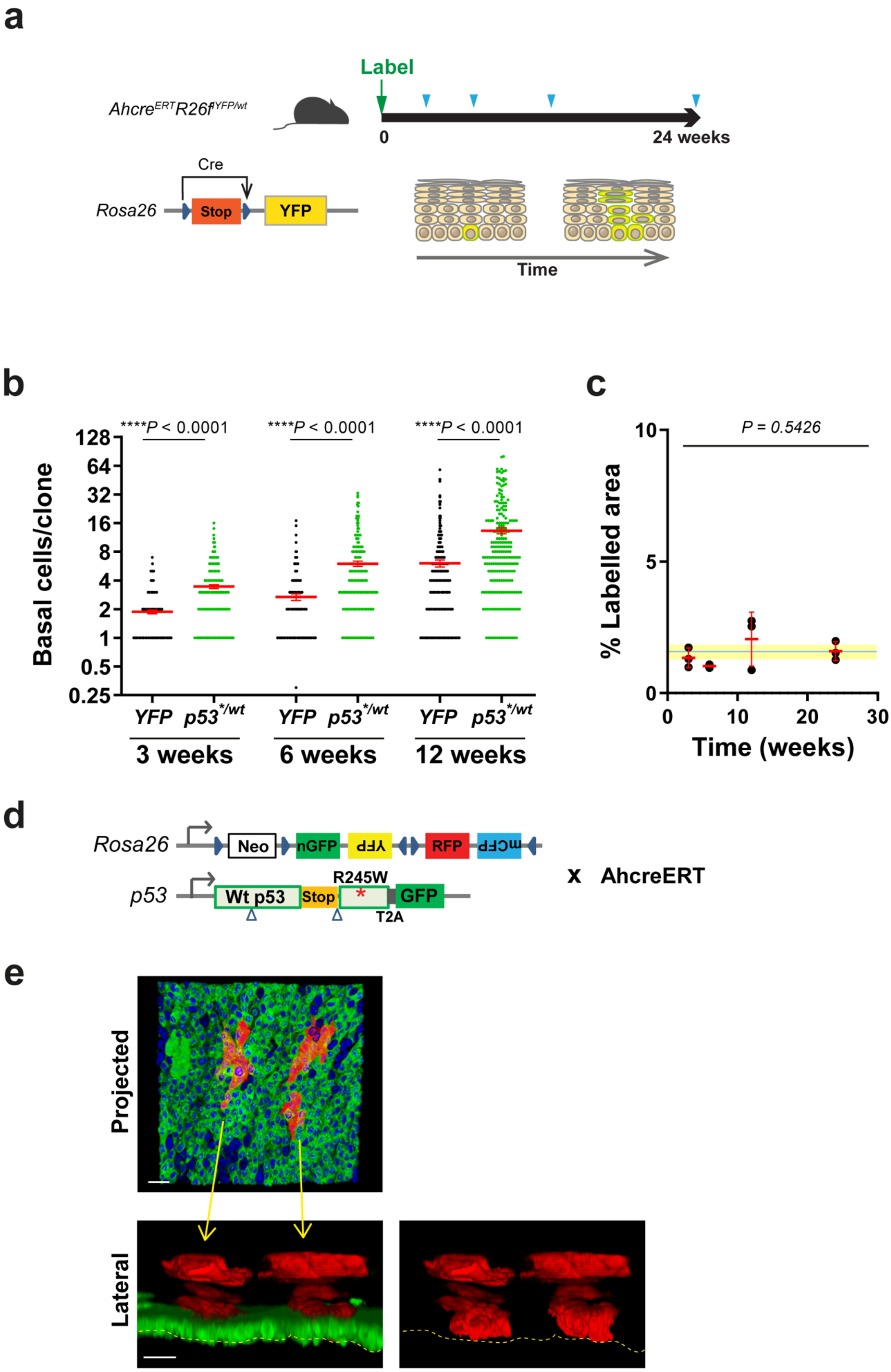

a
Label
$Ahcre^{ERT}R26f^{YFP/wt}$
0
24 weeks
Cre
Rosa26
Stop
YFP
Time
b
****P < 0.0001
****P < 0.0001
****P < 0.0001
Basal cells/clone
128
64
32
16
8
4
2
1
0.5
0.25
YFP
$p53^{*/wt}$
YFP
$p53^{*/wt}$
YFP
$p53^{*/wt}$
3 weeks
6 weeks
12 weeks
c
P = 0.5426
% Labelled area
10
5
0
0
10
20
30
Time (weeks)
d
Rosa26
Neo
nGFP
YFP
RFP
mCFP
x AhcreERT
p53
Wt p53
Stop
R245W
GFP
T2A
e
Projected
Lateral

Supplementary Figure 1: Behaviour of wild type and *p53* mutant clones in homeostatic mouse esophagus. a, Lineage tracing protocol: Clonal frequency labelling was induced in the esophageal epithelium of adult *Ahcre*^{ERT} *R26*$^{flYFP/wt}$ (*p53*$^{wt/wt}$) mice and samples were collected at indicated time points (blue arrowhead). **b,** Comparison of basal clone size. Clone size distribution of *p53*$^{wt/wt}$ (YFP) and *p53*$^{*/wt}$ (GFP) (for ≥2 cell clones). Error bars indicate mean ± s.e.m; 2-4 mice per time point. n=189 YFP and 247 GFP clones at 3 weeks, 140 YFP and 230 GFP clones at 6 weeks, 224 YFP and 223 GFP clones at 12 weeks. *P*-values were determined by two-tailed Mann Whitney test. **c,** Projected labelled area of YFP clones. Error bars indicate mean ± s.e.m. Blue line and shading show average and s.e.m. across all time points. n=11 mice. *p* value was determined by Kruskal-Wallis test. **d,** Multicolour lineage tracing in *Ahcre*^{ERT} *Rosa26*$^{flConfetti/wt}$ *p53*$^{*/wt}$ animals. The fate of *p53* mutant progenitors can be tracked in double labelled clones even if the p53 locus becomes inactive in the differentiated progeny. **e,** Rendered z stacks of typical *Confetti (RFP)-p53*$^{*/wt}$ double labelled clone 12 weeks post induction of *Ahcre*^{ERT} *Rosa26*$^{flConfetti/wt}$ *p53*$^{*/wt}$ animals. Due to the high level of induction, clones were fused by 12 week time point. However double labelled clones could be identified as shown in this image. n = 60 double labelled clones from 2 mice. Red, RFP, Green, GFP, Blue, DAPI. Scale bar, 20 µm. Source data are provided in the Supplementary Data.

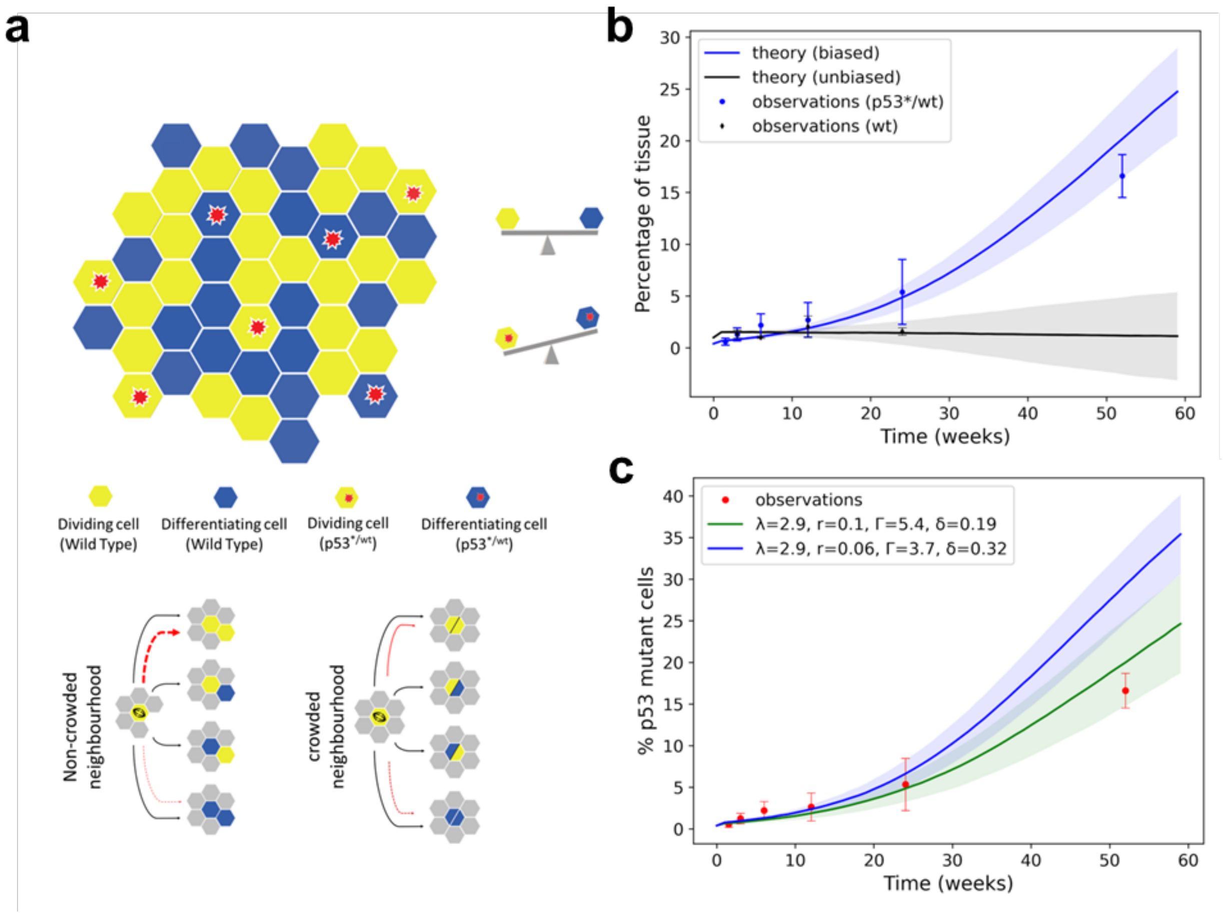

Supplementary Figure 2: **Model of p53$^{*/wt}$ mutant clone dynamics.**The growth of *p53* mutations in the basal layer of the mouse esophageal epithelium is successfully reproduced by a spatial non-neutral SP model with a cell density dependent feedback that mutant cells respond to **a:** Upper panel: Illustration of the two-dimensional hexagonal lattice representing the basal layer. Proliferating cells are shown in yellow and differentiating cells in blue. Proliferating cells divide, producing two daughter cells, whilst differentiating cells exit the basal layer and are removed from the simulation. Mutant cells, marked with a red asterisk, have a bias towards producing proliferating daughters. Lower panel: Schematic representation of the rules of the spatial model. Proliferating cells undergo a division type with balanced division probabilities. In case of mutant cells, division probabilities are biased, favoring symmetric division, indicated by dashed red lines. Mutants lose this advantage in response to crowding in their local neighborhood and switch their behavior to balanced dynamics. **b:** Modelling of tissue colonization. Blue color corresponds to the observed (circles) and simulated (solid line) tissue percentage occupied by mutant cells over time, modelled with a bias in their division outcome probabilities. Black color corresponds to the observed

(diamonds) and simulated (solid line) tissue percentage occupied by a subset of wild-type cells, modelled with balanced division dynamics. Simulated data correspond to mean values across 100 simulations. Shaded areas and error bars correspond to SD. Parameters used: $r = 0.1$, $\Gamma = 3.5$/week, $\lambda = 1.9$/week, from [14]. Fate bias in mutant cells was calculated as $\delta = 0.29$, from a linear regression applied to the In(average clone size), where $2\delta r\lambda$ = slope. Crowding threshold was set to 7 cells, see methods for details. **c:** Analysis of the spatial non-neutral SP model with cell density feedback, simulating *p53* mutant growth in the basal layer of mouse esophageal epithelium under different inferred parameter sets (see methods). Plot showing the simulated (solid lines) and observed (red points) percentage of mutant cells over time. Simulated data correspond to mean values across 100 simulations. Shaded areas and error bars correspond to SD. The values of r, Γ, λ were taken from[16]. The value of fate bias (δ) was calculated from a linear regression applied to the In(average clone size), where $2\delta r\lambda$ = slope.

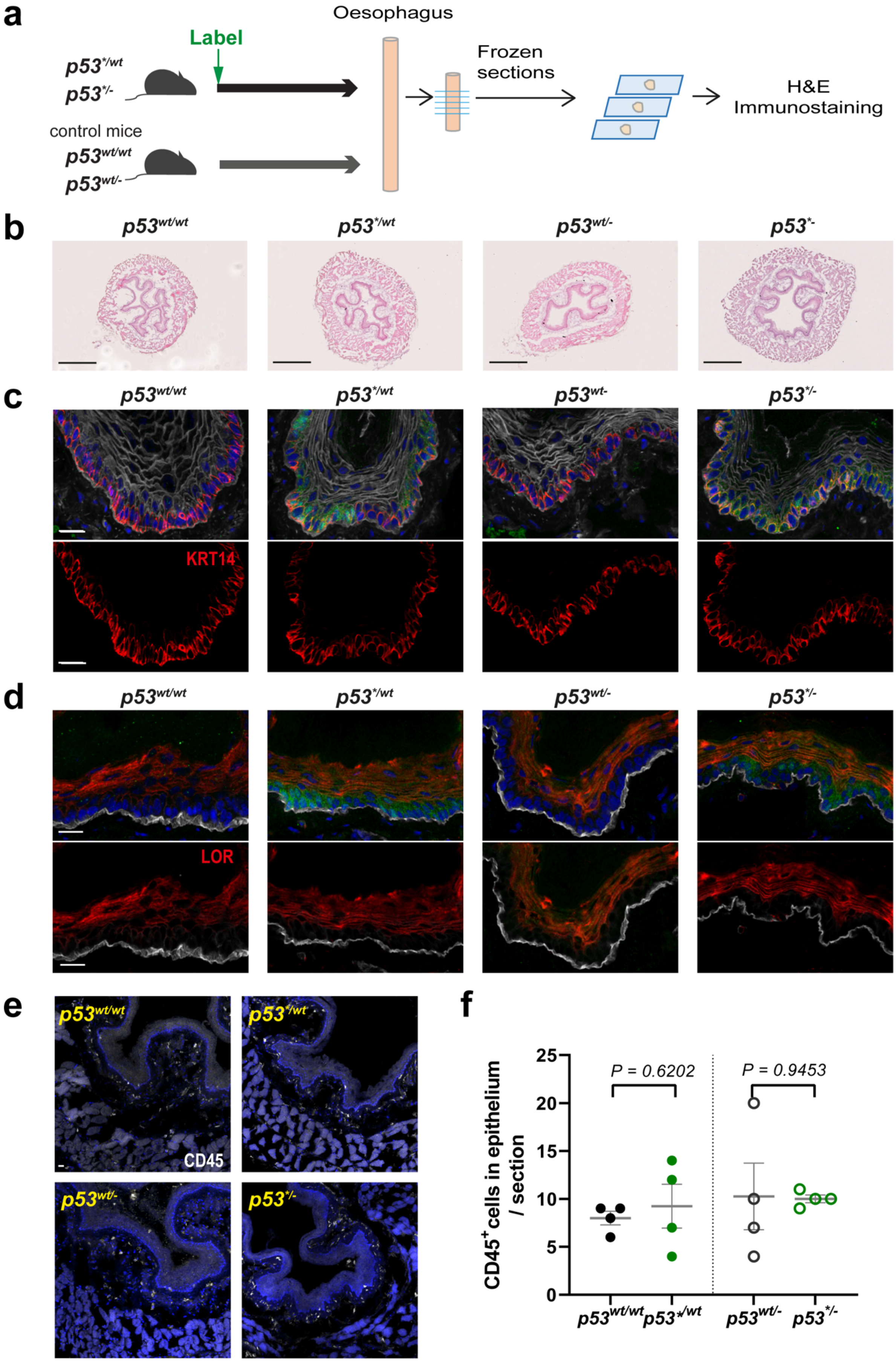
a
Oesophagus
Label
p53*/wt
p53*/-
control mice
p53wt/wt
p53wt/-
Frozen
sections
H&E
Immunostaining
b
p53wt/wt
p53*/wt
p53wt/-
p53*-
c
p53wt/wt
p53*/wt
p53wt-
p53*/-
KRT14
d
p53wt/wt
p53*/wt
p53wt/-
p53*/-
LOR
e
p53wt/wt
p53*/wt
CD45
p53wt/-
p53*/-
f
CD45+cells in epithelium / section
P = 0.6202
P = 0.9453
0
5
10
15
20
25
p53wt/wt
p53*/wt
p53wt/-
p53*/-

Supplementary Figure 3: Effect of *p53 mutation on esophageal epithelium. a,** Protocol: Cryosections of mouse esophageal epithelium from mice with indicated genotypes were generated as shown, $p53^{*/wt}$ and $p53^{*/-}$ esophagus was harvested at 8 months post high level induction. **b-e,** Representative images from n=3 mice per group. **b,** H and E. Scale bars, 500µm. **c-e,** Blue, DAPI. Scale bar, 20µm. **c,** Basal layer, KRT14 (red). Green, GFP; grey, WGA. **d,** Differentiated cells in suprabasal layer, LOR (red). Green, GFP; grey, ITGA6. **e,** Pan leukocyte marker, CD45 (grey). **f** Number of CD45+ cells in epithelium per transverse section, n=4 sections from 2 mice per genotype. Error bars indicate mean ± s.e.m. *p* value determined by two tailed unpaired t-test. Source data are shown in Supplementary Data.

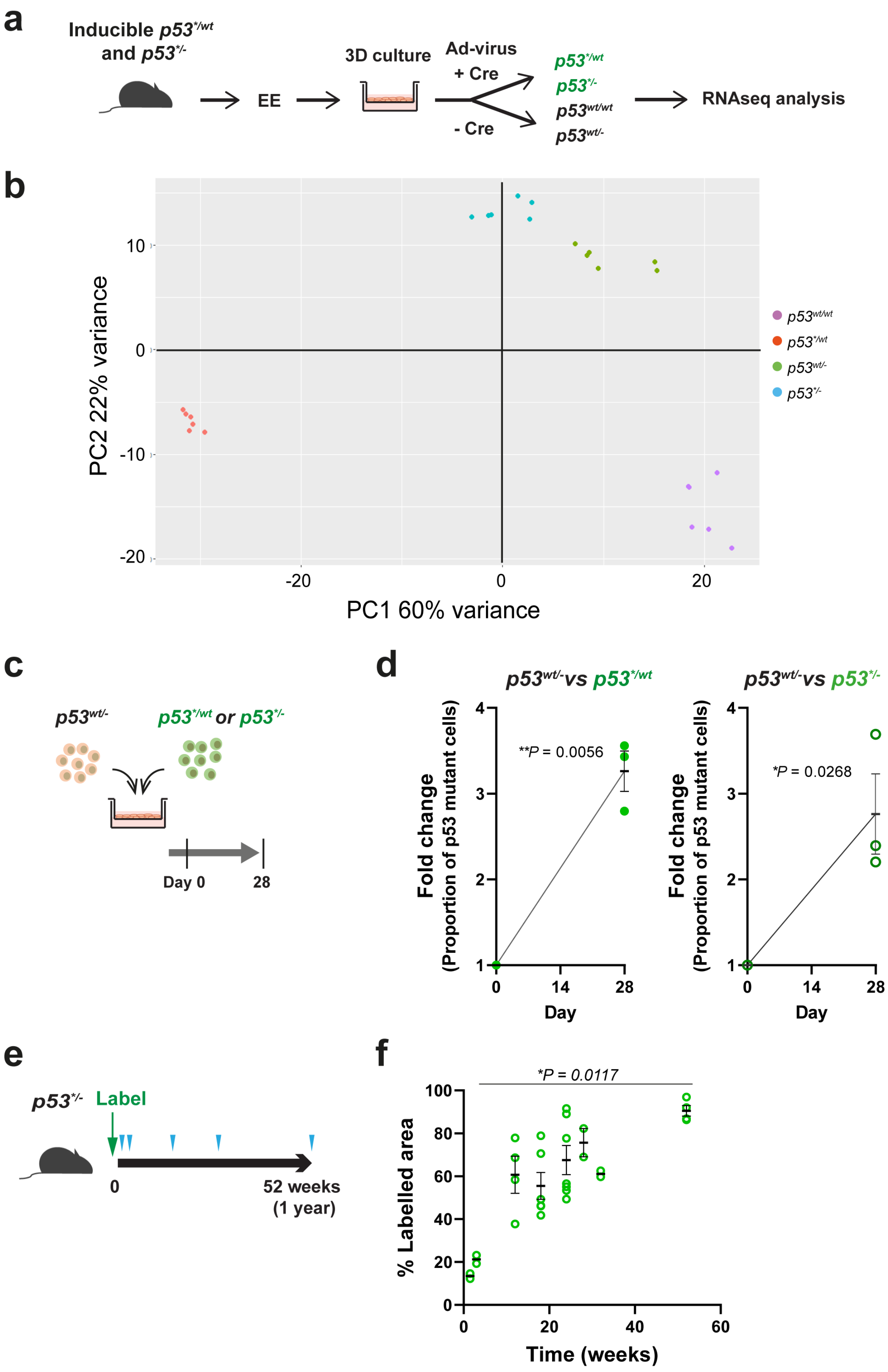
a
Inducible p53*/wt and p53*/-
EE
3D culture
Ad-virus + Cre
- Cre
p53*/wt
p53*/-
p53wt/wt
p53wt/-
RNAseq analysis
b
PC2 22% variance
PC1 60% variance
10
0
-10
-20
20
p53wt/wt
p53*/wt
p53wt/-
p53*/-
c
p53wt/-
p53*/wt or p53*/-
Day 0
28
d
p53wt/- vs p53*/wt
p53wt/- vs p53*/-
Fold change (Proportion of p53 mutant cells)
**P = 0.0056
*P = 0.0268
1
2
3
4
0
14
28
Day
e
p53*/-
Label
0
52 weeks (1 year)
f
*P = 0.0117
% Labelled area
100
80
60
40
20
0
Time (weeks)

Supplementary Figure 4: Characterization of *p53* mutant cells. a, Primary esophageal keratinocytes from transgenic mice were culture in 3D and *p53** mutation was induced using adenovirus encoding Cre recombinase. **b,** Principal component analysis of RNAseq data showing the variation of gene expression profile between samples. Total RNA was collected from the primary cultured esophageal keratinocytes of indicated genotype and RNAseq analysis was carried out. **c,** Protocol: $p53^{*/wt}$ and $p53^{*/-}$ cells were cocultured with $p53^{wt/-}$ cells respectively and relative fitness was examined. **d,** Quantitation of cell competition assay by flow cytometry. Graph shows the fold change of proportion of GFP+ $p53^{*/wt}$ or $p53^{*/-}$ cell in the culture. Black ($p53^{*/wt}$) and red ($p53^{*/-}$) lines indicate mean and s.e.m. *p* value was determined by two-tailed ratio paired t-test, n= 3 biological replicate cultures. **e,** *in vivo* analysis of $p53^{*/-}$ clone behaviour. Expression of *p53* mutant and GFP reporter was induced clonally (Labelling) and esophagus samples were taken at indicated time points (triangles). The fate of mutant clones was examined by tracking the expression of GFP. **f,** Proportion of labelled projected area at indicated time points. Error bars are mean ± s.e.m. n=2 mice for 1.5, 3, 28 and 32 week time points, n=4 for 12 week, n=6 for 18 week, n=7 for 24 week and n=4 for 52 week time points. *p* value was determined by Kruskal-Wallis test. Source data are provided in Supplementary Data.

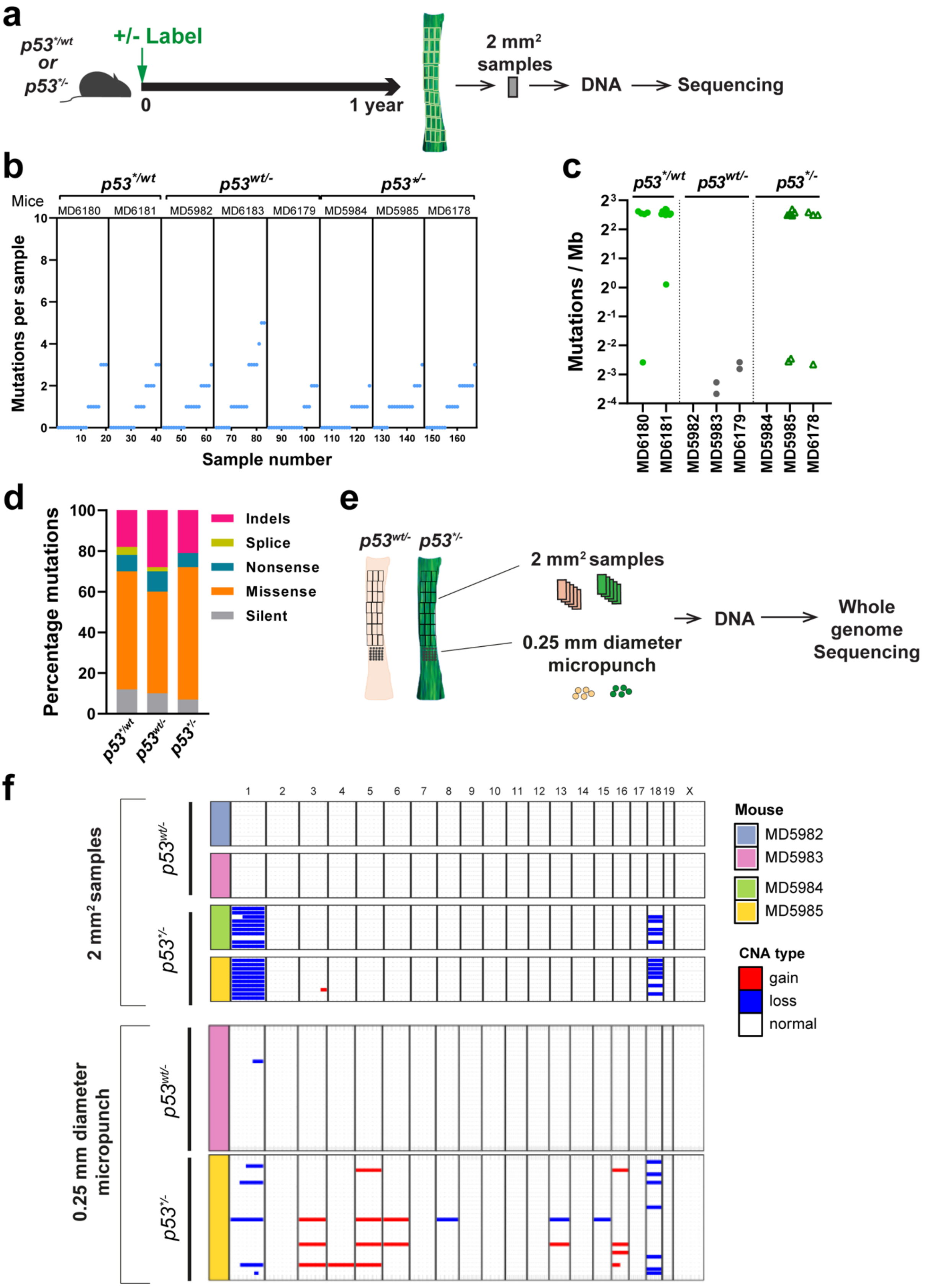
a
p53*/wt
or
p53*/-
+/- Label
0
1 year
2 mm² samples
DNA
Sequencing
b
p53*/wt
p53wt/-
p53*/-
Mice
MD6180
MD6181
MD5982
MD6183
MD6179
MD5984
MD5985
MD6178
Mutations per sample
Sample number
c
p53*/wt
p53wt/-
p53*/-
Mutations / Mb
MD6180
MD6181
MD5982
MD5983
MD6179
MD5984
MD5985
MD6178
d
Percentage mutations
Indels
Splice
Nonsense
Missense
Silent
p53*/wt
p53wt/-
p53*/-
e
p53wt/-
p53*/-
2 mm² samples
0.25 mm diameter micropunch
DNA
Whole genome Sequencing
f
2 mm² samples
0.25 mm diameter micropunch
p53wt/-
p53*/-
Mouse
MD5982
MD5983
MD5984
MD5985
CNA type
gain
loss
normal

Supplementary Figure 5: DNA sequencing analysis of *p53* mutant EE. a, Protocol: EE from indicated mice was collected at 1 year time point and cut into 2mm^2 grid pieces. DNA from each sample was analysed by ultradeep targeted sequencing (a-d) or WGS (e). **b,** Number of mutations per sample. Every dot corresponds to a sample. n=2 mice for $p53^{*/wt}$ and n=3 mice for $p53^{wt/-}$ and $p53^{*/-}$**. c,** Estimated mutation burden in each sample. No significant difference between genotypes by two tailed unpaired Student's or Welch's t-test. **d,** Percentage of mutation types identified in each genotype. **e,** Copy number analysis comparing sample size. 2 mm^2 and micro-punch biopsies were taken from same EE and subjected to whole genome sequencing. **f,** Summary of copy number analysis. The result with 2 mm^2 samples was comparable to that detected by ultradeep targeted sequencing (**Fig. 4h**), whereas micro-punch biopsies showed more variety of copy number alterations. Source data are provided in Supplementary Data.

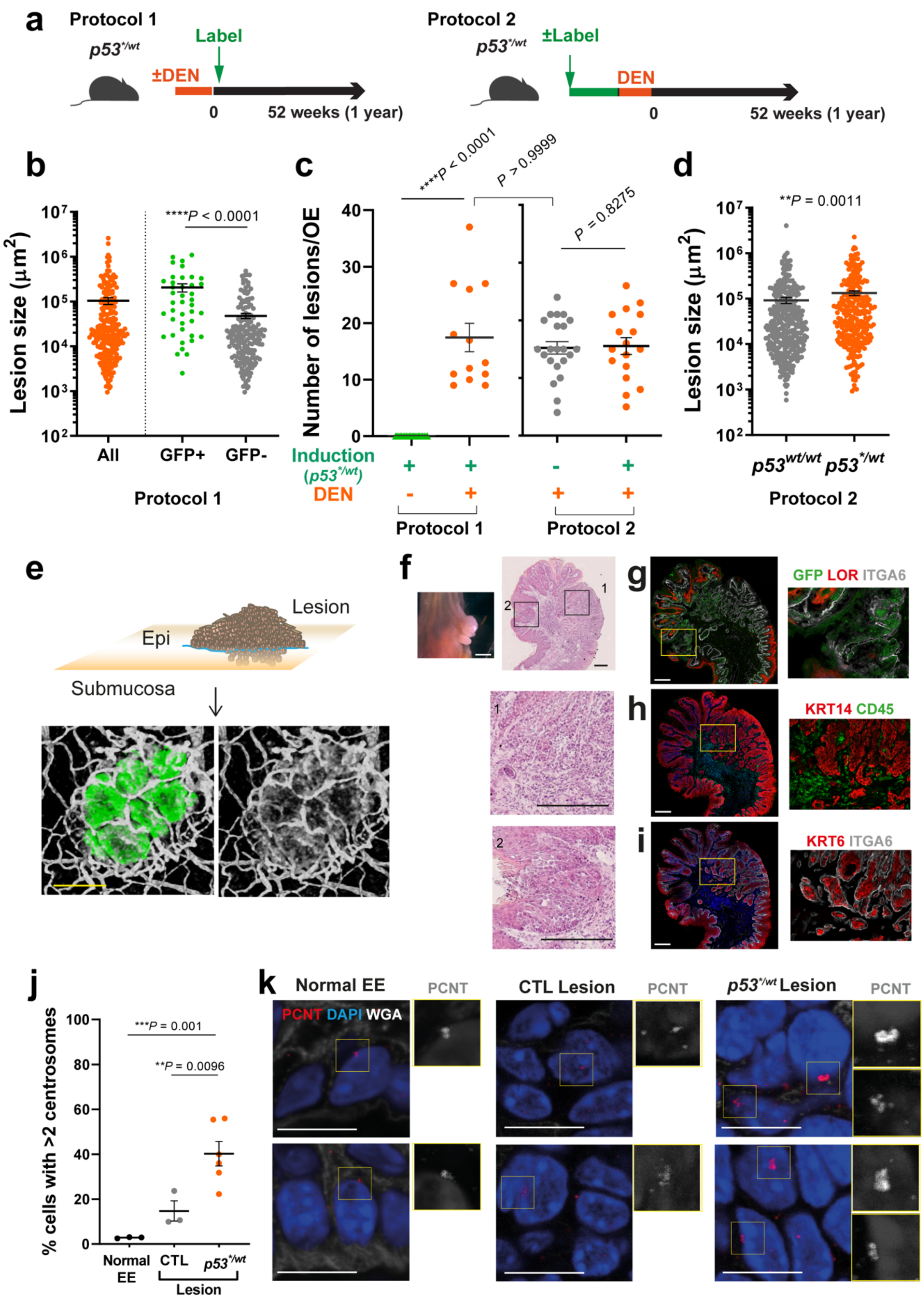
a
Protocol 1
p53*/wt
Label
±DEN
0
52 weeks (1 year)
Protocol 2
p53*/wt
±Label
DEN
0
52 weeks (1 year)
b
Lesion size (μm²)
****P < 0.0001
All
GFP+
GFP-
Protocol 1
c
****P < 0.0001
P > 0.9999
P = 0.8275
Number of lesions/OE
Induction (p53*/wt)
DEN
Protocol 1
Protocol 2
d
**P = 0.0011
Lesion size (μm²)
p53wt/wt
p53*/wt
Protocol 2
e
Lesion
Epi
Submucosa
f
g
GFP LOR ITGA6
h
KRT14 CD45
i
KRT6 ITGA6
j
% cells with >2 centrosomes
***P = 0.001
**P = 0.0096
Normal EE
CTL
p53*/wt
Lesion
k
Normal EE
PCNT
CTL Lesion
PCNT
p53*/wt Lesion
PCNT
PCNT DAPI WGA

Supplementary Figure 6: Lesions formed in mutagenized epithelium carrying *p53*$^{*/wt}$ clones. **a,** Protocol 1: *p53** mutation was induced after DEN treatment, *p53*$^{*/wt}$ clones competed in mutated EE, less than 10% of them persisted. Protocol 2: Induced *p53*$^{*/wt}$ mice were treated with DEN, introducing mutations within *p53*$^{*/wt}$ cells which had expanded to occupy >70% of the epithelium. Lesions found in these mice between 24 and 52 weeks (time determined by when mice were culled for welfare reasons) were quantified. **b,** Comparison of lesion size in protocol 1. Error bars indicate mean ± s.e.m.; two-tailed Mann Whitney test. n=227 lesions from 13 mice. Contribution of *p53*$^{*/wt}$ to the lesions was also assessed by GFP expression (GFP+). **c,** Number of lesions per oesophagus (OE). Error bars indicate mean ± s.e.m. n=22 for induced *p53*$^{*/wt}$ control (untreated), n=13 for DEN-treated induced mice, n=22 for uninduced mice (*p53*$^{wt/wt}$*)* and n=17 for *p53*$^{*/wt}$. *p* values were determined by two-tailed Mann Whitney test. **d,** Comparison of lesion size in protocol 2. The area of lesions was recorded at late time points. Error bars indicate mean ± s.e.m; two-tailed Mann Whitney test. n=335 lesions from 22 mice for *p53*$^{wt/wt}$ and n=264 lesions from 17 mice for *p53*$^{*/wt}$. **e,** Schematic of lesion and 3D confocal images of submucosa showing invasion of epithelial cells. Esophageal samples were collected from *p53*$^{*/wt}$ induced mice at 6 months post DEN treatment. After the epithelium was peeled off, the submucosa was stained for GFP (*p53*$^{*/wt}$, green), KRT6 (red) and CD31 (grey). The dotted yellow boxes are shown at higher magnification. n = 15 tumors from 2 mice. Scale bars, 200μm. **f,** Image of ESCC and histology. n=2 confirmed ESCC from 16 tumors. White scale bar 1mm, black scale bars, 200 μm. **g,** GFP for transgenic *p53* mutant expression; LOR, differentiated keratinocyte; ITGA6, basal membrane. **h,** KRT14, progenitors in basal layer; CD45, leukocytes. **i,** KRT6, hyperplastic epithelium marker; ITGA6. **g-i,** Scale bars, 200μm. **j,** Quantitation of centrosome per cell in macroscopic lesions at one year time point. Frozen sections of macroscopic lesions were stained with PCNT (**Supplementary Figure 6k**). Centrosomes were quantified based on number of PCNT foci and size (area). Values are the average number of centrosomes per cell from each normal EE or lesion. Error bars indicate mean ± s.e.m; P-values were determined by two-tailed unpaired t test with Welch's correction. **j-k**, n=299 cells from 3 normal mouse EE, n=332 cells from 3 control (CTL) lesions (n=2 mice), n=614 cells from 6 *p53*$^{*/wt}$ lesions (n=3 mice). **k,** Representative images of centrosomes in macroscopic lesions. Frozen section of normal EE (untreated), CTL (*p53*$^{wt/wt}$) and *p53*$^{*/wt}$ lesions were stained for PCNT. Red, PCNT; glue, DAPI; grey, WGA. Enlarged PCNT foci are shown in greyscale. Scale bars, 10μm. Source data are provided in Supplementary Data.

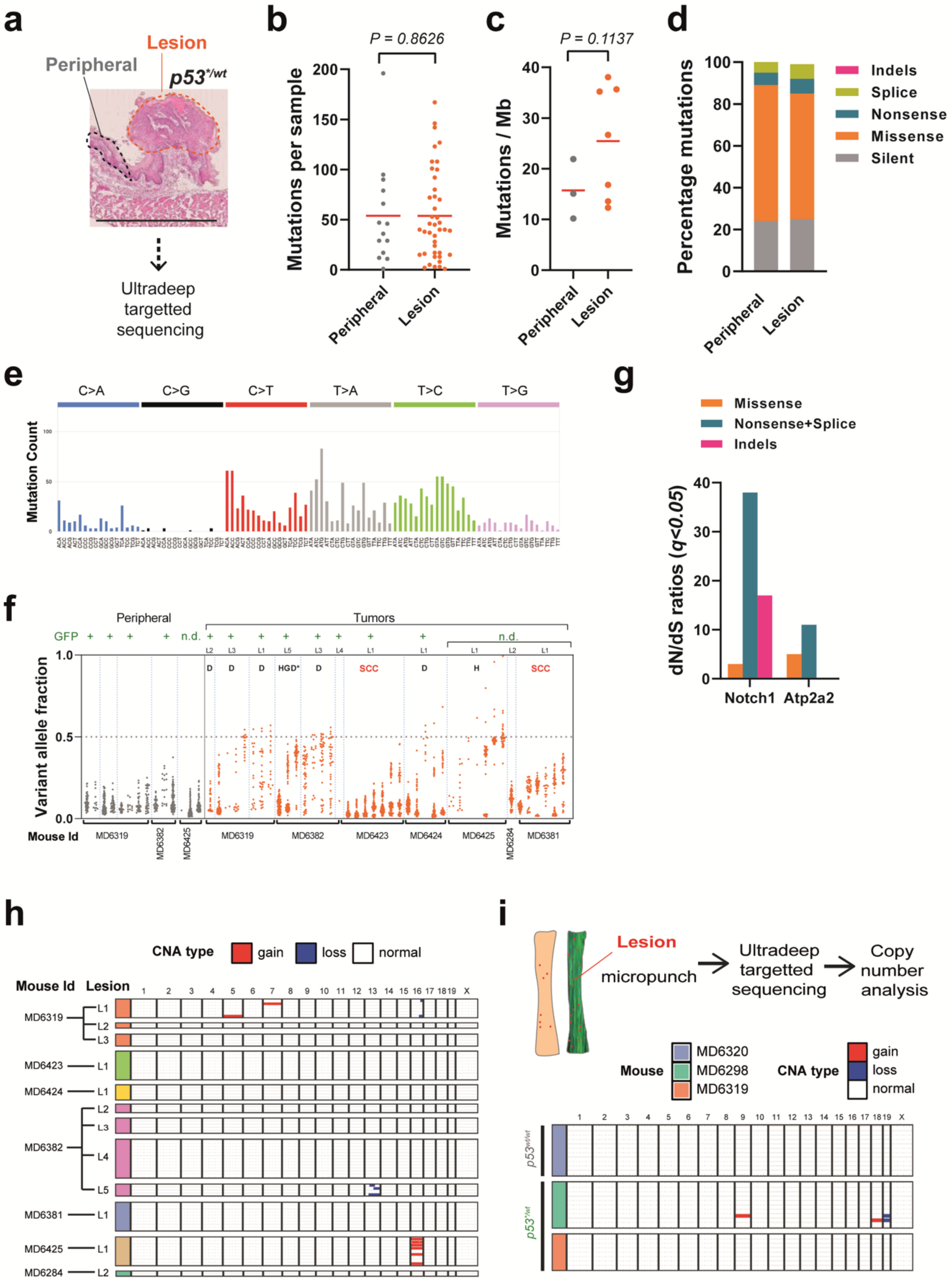
a
Lesion
Peripheral
p53*/wt
Ultradeep targetted sequencing
b
P = 0.8626
Mutations per sample
Peripheral
Lesion
c
P = 0.1137
Mutations / Mb
d
Percentage mutations
Indels
Splice
Nonsense
Missense
Silent
e
C>A
C>G
C>T
T>A
T>C
T>G
Mutation Count
f
Peripheral
Tumors
GFP
n.d.
Variant allele fraction
SCC
HGD*
Mouse Id
MD6319
MD6382
MD6425
MD6423
MD6424
MD6284
MD6381
g
Missense
Nonsense+Splice
Indels
dN/dS ratios (q<0.05)
Notch1
Atp2a2
h
CNA type
gain
loss
normal
Mouse Id
Lesion
i
micropunch
Ultradeep targetted sequencing
Copy number analysis
Mouse
MD6320
MD6298
MD6319
CNA type

Supplementary Figure 7: Mutational landscape and CNA in *p53*$^{*/wt}$ lesions. a, Sequencing samples: macroscopic esophageal lesions from DEN treated *p53*$^{*/wt}$ mice were sectioned and stained with H&E. Lesions and peripheral areas were scraped off from slides and DNA was extracted for ultradeep targeted sequencing. n=42 tumour sections from 7 mice. **b,** Number of mutations per sample. Each dot corresponds to a sample. Red lines indicate mean value. *p* value was determined by two-tailed Mann Whitney test. n=14 peripheral area from 3 mice and n=42 tumour sections from 7 mice. **c,** Estimated mutation burden. Each dot represents a mouse. Red lines indicate mean value. *p* value was determined by two-tailed unpaired t test with Welch's correction. n=3 mice for peripheral and n=7 mice for tumour samples. **d,** Percentage of mutation types identified in each group. **e,** Mutational spectrum of lesions. **f,** Variant allele fraction for the mutations detected in samples. Samples from the same lesion were grouped. Pathology result of lesions were indicated in the panel: D, dysplasia; HGD*, high grade dysplasia with potential invasion; H, hyperplasia; SCC, squamous cell carcinoma. GFP expression was determined where possible and indicated at the top of the panel. **g,** Positively selected somatic mutations in tumors. dN/dS ratios for missense, truncating (nonsense + splice) and indels of mutant genes under selection are shown. **h,** CNA in macroscopic tumors. n = 12 tumors from 7 mice. **i,** CNA in microscopic tumors. 18 tumors from 2 induced mice and 11 tumors from uninduced mouse were analysed by off target reads from ultradeep targeted sequencing, see methods. Source data are provided in Supplementary Data.

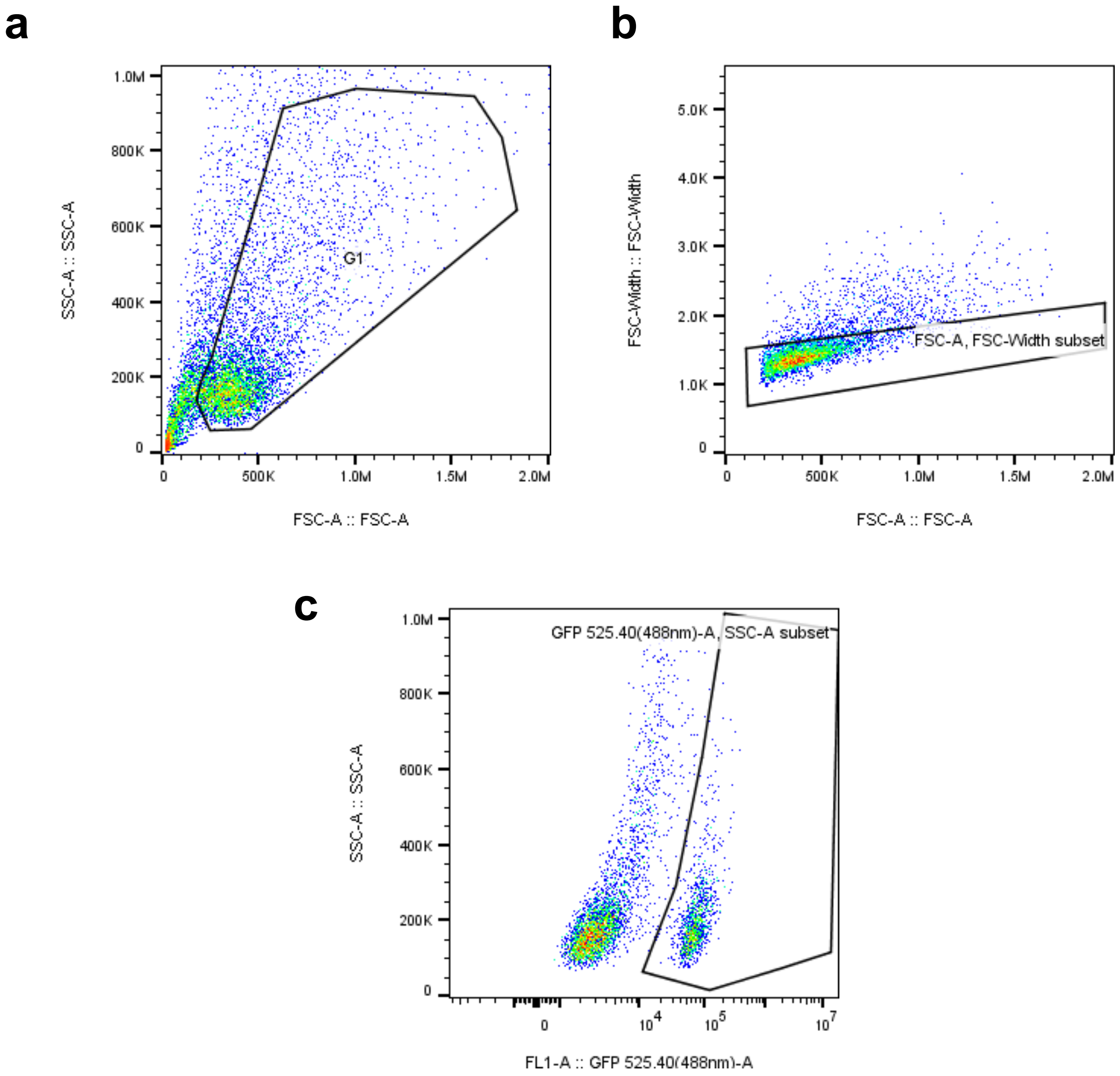

Supplementary Figure 8: Flow cytometry gating strategy FSC/SSC gating was applied to identify cell population of interest and to exclude debris (**a**). Doublet and multiplet cells were excluded by FSC-A/FSC-Width gating (**b**). Boundaries of GFP positive and negative population are defined using control samples (**c**).
