# Supplementary material for: p53 mutation in normal esophagus promotes multiple stages of carcinogenesis but is constrained by clonal competition
Source: Nat Commun. 2022 Oct 20;13:6206. doi: 10.1038/s41467-022-33945-y (PMC9584949; doi:10.1038/s41467-022-33945-y)
Supplement: Supplementary file 3 — Reporting Summary [file 41467_2022_33945_MOESM3_ESM.pdf]

nature portfolio

Corresponding author(s): Philip H Jones
Last updated by author(s): Sep 21, 2022

Reporting Summary

Nature Portfolio wishes to improve the reproducibility of the work that we publish. This form provides structure for consistency and transparency in reporting. For further information on Nature Portfolio policies, see our Editorial Policies and the Editorial Policy Checklist.

Statistics

For all statistical analyses, confirm that the following items are present in the figure legend, table legend, main text, or Methods section.

n/a	Confirmed	
[ ]	[x]	The exact sample size (*n*) for each experimental group/condition, given as a discrete number and unit of measurement
[ ]	[x]	A statement on whether measurements were taken from distinct samples or whether the same sample was measured repeatedly
[ ]	[x]	The statistical test(s) used AND whether they are one- or two-sided<br>*Only common tests should be described solely by name; describe more complex techniques in the Methods section.*
[ ]	[x]	A description of all covariates tested
[ ]	[x]	A description of any assumptions or corrections, such as tests of normality and adjustment for multiple comparisons
[ ]	[x]	A full description of the statistical parameters including central tendency (e.g. means) or other basic estimates (e.g. regression coefficient) AND variation (e.g. standard deviation) or associated estimates of uncertainty (e.g. confidence intervals)
[ ]	[x]	For null hypothesis testing, the test statistic (e.g. *F*, *t*, *r*) with confidence intervals, effect sizes, degrees of freedom and *P* value noted<br>*Give P values as exact values whenever suitable.*
[x]	[ ]	For Bayesian analysis, information on the choice of priors and Markov chain Monte Carlo settings
[x]	[ ]	For hierarchical and complex designs, identification of the appropriate level for tests and full reporting of outcomes
[x]	[ ]	Estimates of effect sizes (e.g. Cohen's *d*, Pearson's *r*), indicating how they were calculated

Our web collection on statistics for biologists contains articles on many of the points above.

Software and code

Policy information about availability of computer code

Data collection	Imaging: LAX X (v3.5.7.23225, Leica)
Data analysis	RNA seq analysis: R (v3.5.1, https://www.R-project.org/), with the packages pheatmap (v1.0.10, https://CRAN.R-project.org/package=pheatmap), clusterProfiler (v3.8.1, https://www.bioconductor.org/packages/devel/bioc/html/clusterProfiler.html)39 with mouse annotation provided by org.Mm.eg.db (v3.6.0, http://bioconductor.org/packages/org.Mm.eg.db/). Heatmaps were generated from the Transcript Per Million (TPM) values using Morpheus web analysis tool from Broad Institute (https://software.broadinstitute.org/morpheus/).<br><br>DNA sequencing: BWA-MEM (v.0.7.16a & v0.7.17, https://github.com/lh3/bwa), Biobambam2 (v.2.0.86, https://gitlab.com/german.tischler/biobambam2), deepSNV package (v1.21.3, https://github.com/gerstung-lab/deepSNV), CaVEMan (v1.13.14 & v1.14.0) and Pindel (v3.3.0), VAGrENT (v3.7.0), dNdScv algorithm (v0.0.1.0, https://github.com/im3sanger/dndscv), R package (v1.9.0, https://github.com/raerose01/deconstructSigs), MutationalPatterns (v3.4.0, https://bioconductor.org/packages/release/bioc/html/MutationalPatterns.html), QDNAseq (https://github.com/ccagc/QDNAseq/)<br><br>The pipeline code and modified QDNAseq package (https://github.com/sdentro/qdnaseq_pipeline and https://github.com/sdentro/QDNAseq/tree/dev)<br><br>Imaging: Volocity v6 and v6.3<br>Statistics: Graphpad Prism v8.3.1<br><br>Flow cytometry:<br>CytoExpert (Beckman Coulter Life Sciences)<br>FlowJo v10.6.1 (Becton Dickinson)

For manuscripts utilizing custom algorithms or software that are central to the research but not yet described in published literature, software must be made available to editors and reviewers. We strongly encourage code deposition in a community repository (e.g. GitHub). See the Nature Portfolio guidelines for submitting code & software for further information.

Data

Policy information about availability of data

All manuscripts must include a data availability statement. This statement should provide the following information, where applicable:
- Accession codes, unique identifiers, or web links for publicly available datasets
- A description of any restrictions on data availability
- For clinical datasets or third party data, please ensure that the statement adheres to our policy

The sequencing data sets in this study are available at the European Nucleotide archive (ENA) Accession numbers for RNAseq data on https://www.ebi.ac.uk/ena are as follows: p53wt/wt, ERS1432686, ERS1432689, ERS1432688, ERS1432703, ERS1432704, ERS1432705; p53*/wt , ERS1432636, ERS1432637, ERS1432634, ERS1432638, ERS1432635, ERS1432639; p53wt/-, ERS1432650, ERS1432651, ERS1432656, ERS1432654, ERS1432657, ERS1432658; p53*/-, ERS1432672, ERS1432670, ERS1432671, ERS1432673, ERS1432674, ERS1432676.

Accession numbers for targeted DNA sequencing samples and Whole genome sequencing are ERP129331 and ERP129332 respectively.

GRCm38 mouse genome:GCA_000001635.8, GCF_000001635.26

Field-specific reporting

Please select the one below that is the best fit for your research. If you are not sure, read the appropriate sections before making your selection.

☒ Life sciences ☐ Behavioural & social sciences ☐ Ecological, evolutionary & environmental sciences

For a reference copy of the document with all sections, see nature.com/documents/nr-reporting-summary-flat.pdf

Life sciences study design

All studies must disclose on these points even when the disclosure is negative.

Sample size	Sample size was not predetermined by statistical methods. Sampling size was determined by pilot studies for lineage tracing and clonal sequencing studies and by previously published studies for highly mutagenized sequencing and carcinogenesis studies.<br>Doupe, D. P. et al. A single progenitor population switches behavior to maintain and repair esophageal epithelium. Science 337, 1091-1093, (2012).<br>Alcolea, M. P. et al. Differentiation imbalance in single oesophageal progenitor cells causes clonal immortalization and field change. Nat Cell Biol 16, 615-622, (2014).<br>Fernandez-Antoran, D. et al. Outcompeting p53-Mutant Cells in the Normal Esophagus by Redox Manipulation. Cell stem cell 25, 329-341, (2019).<br>Frede, J., Greulich, P., Nagy, T., Simons, B. D. & Jones, P. H. A single dividing cell population with imbalanced fate drives oesophageal tumour growth. Nat Cell Biol 18, 967-978, (2016).<br>Colom, B. et al. Mutant clones in normal epithelium outcompete and eliminate emerging tumours. Nature 598, 510-514, (2021).<br>Colom, B. et al. Spatial competition shapes the dynamic mutational landscape of normal esophageal epithelium. Nature Genetics 52, 604–614, (2020).
Data exclusions	No data were excluded from the analysis.
Replication	Biological replicates were performed in all cases, numbers of replicates are given in figure legends and source data tables.
Randomization	Allocation of individual mice of the required genotype was random.
Blinding	Blinding was not relevant to planning of experiments since mouse models in this study required genotype assessment. The investigator was blinded to group allocation for DEN treatment and sampling time points. p53 mutant samples express GFP which is not present in controls, therefore blinding was not possible during imaging analysis.

Reporting for specific materials, systems and methods

We require information from authors about some types of materials, experimental systems and methods used in many studies. Here, indicate whether each material, system or method listed is relevant to your study. If you are not sure if a list item applies to your research, read the appropriate section before selecting a response.

Materials & experimental systems

n/a	Involved in the study
	☒ Antibodies
☒	Eukaryotic cell lines
☒	Palaeontology and archaeology
	☒ Animals and other organisms
☒	Human research participants
☒	Clinical data
☒	Dual use research of concern

Methods

n/a	Involved in the study
☒	ChIP-seq
	☒ Flow cytometry
☒	MRI-based neuroimaging

Antibodies

Antibodies used

anti-GFP/YFP (ThermoFisher Scientific, A10262), anti-Cytokeratin 14 (Covance, PRB-155P), anti-Cytokeratin 6 (Biolegend, 905701), anti-pericentrin (abcam, ab4448), anti-CD45 (Biolegend, 103102), anti-CD31 (abcam, ab7388), anti-Loricrin (Covance PRB-145P), Alexa Fluor 647 anti-CD49f (Biolegend, 313610).

Secondary antibodies: 488 anti-chicken (Jackson ImmunoResearch, 703-545-155), 555 anti-rabbit (ThermoFisher Scientific, A31572), 647 anti-mouse (ThermoFisher Scientific, A32787), 488 and 647 anti-rat (ThermoFisher Scientific, A21208 and A48272)

Validation

All antibodies were validated in mouse and the techniques used in the study according to the manufacture's website and in previous publications.
anti-GFP/YFP (ThermoFisher Scientific, A10262)
https://www.thermofisher.com/antibody/product/GFP-Antibody-Polyclonal/A10262
anti-Cytokeratin 14 (Covance, PRB-155P)
https://www.biolegend.com/Files/Images/media_assets/pro_detail/datasheets/905301_V04_051117.pdf
anti-Cytokeratin 6 (Biolegend, 905701)
https://www.biolegend.com/fr-ch/products/purified-anti-mouse-keratin-6a-antibody-11459?pdf=true&displayInline=true&leftRightMargin=15&topBottomMargin=15&filename=Purified%20anti-mouse%20Keratin%206A%20Antibody.pdf&v=20220606063221
anti-pericentrin (abcam, ab4448)
https://www.abcam.com/pericentrin-antibody-centrosome-marker-ab4448.html
anti-CD45 (Biolegend, 103102)
https://www.biolegend.com/fr-ch/products/purified-anti-mouse-cd45-antibody-102
anti-CD31 (abcam, ab7388)
https://www.abcam.com/cd31-antibody-mec-746-ab7388.html
anti-Loricrin (Covance PRB-145P)
https://www.biolegend.com/Files/Images/media_assets/pro_detail/datasheets/905101_V04_051117.pdf
Alexa Fluor 647 anti-CD49f (Biolegend, 313610)
https://www.biolegend.com/it-it/products/alexa-fluor-647-anti-human-mouse-cd49f-antibody-3290

anti-GFP/YFP, anti-GFP/YFP, anti-Cytokeratin 6, anti-CD45, anti-CD31, anti-Loricrin, Alexa Fluor 647 anti-CD49f and secondary antibodies were also used as described in the following previous publications:
Fernandez-Antoran, D. et al. Outcompeting p53-Mutant Cells in the Normal Esophagus by Redox Manipulation. Cell stem cell 25, 329-341, (2019).
Murai, K. et al. Epidermal Tissue Adapts to Restrain Progenitors Carrying Clonal p53 Mutations. Cell stem cell 23, 687-699.e688, (2018).
Colom, B. et al. Mutant clones in normal epithelium outcompete and eliminate emerging tumours. Nature 598, 510-514, (2021).
Colom, B. et al. Spatial competition shapes the dynamic mutational landscape of normal esophageal epithelium. Nature Genetics 52, 604–614, (2020).
Frede, J., Greulich, P., Nagy, T., Simons, B. D. & Jones, P. H. A single dividing cell population with imbalanced fate drives oesophageal tumour growth. Nat Cell Biol 18, 967-978, (2016).

Animals and other organisms

Policy information about studies involving animals; ARRIVE guidelines recommended for reporting animal research

Laboratory animals

All mouse strains were maintained on a C57/Bl6 genetic background, specific and opportunistic pathogen free health status and were immune competent.

Strains: AhcreERTRosaflEYFP/wt, AhcreERTRosa26flconfetti/wt, AhcreERTTrp53flR245W-GFP/wt and AhcreERTTrp53flR245W-GFP/-

Mice were housed in individually ventilated cages (at 19-23°C, RH55%±10%, 12/12 light dark cycle, 15-20 air changes per hour) and fed on standard chow. Adult mice were used for in vivo experiments and no animals were involved in previous experiments and were drug naive prior to the start of experiments. Both male and female animals at 11-16 weeks of age at the start of experiments were used in this study.

Wild animals	The study did not involve wild animals.
Field-collected samples	The study did not involve samples collected from the fields.
Ethics oversight	Experiments were conducted according to UK government Home Office project licenses PPL22/2282, PPL70/7543 and PPL4639B40.

Note that full information on the approval of the study protocol must also be provided in the manuscript.

Flow Cytometry

Plots

Confirm that:

- [x] The axis labels state the marker and fluorochrome used (e.g. CD4-FITC).
- [x] The axis scales are clearly visible. Include numbers along axes only for bottom left plot of group (a 'group' is an analysis of identical markers).
- [x] All plots are contour plots with outliers or pseudocolor plots.
- [x] A numerical value for number of cells or percentage (with statistics) is provided.

Methodology

Sample preparation	Primary oesohageal mouse keratinocyte culture was trypsinized, spun and resuspeded in HBSS containing 2%FBS.
Instrument	CytoFLEX S (Beckman Coulter Life Sciences)
Software	CytoExpert (Beckman Coulter Life Sciences)<br>FlowJo v10.6.1 (Becton Dickinson)
Cell population abundance	Total of 10,000 cells events were acquired.
Gating strategy	FSC/SSC gating was applied to identify cell population of interest and to exclude debris. Doublet and multiplet cells were excluded by FSC-A/FSC-Width gating. Boundaries of GFP positive and negative population are defined using control samples (GFP expressing or non-expressing control). This is shown in Supplementary Figure 8.

- [x] Tick this box to confirm that a figure exemplifying the gating strategy is provided in the Supplementary Information.
