# Supplementary material for: p53 mutation in normal esophagus promotes multiple stages of carcinogenesis but is constrained by clonal competition
Source: Nat Commun. 2022 Oct 20;13:6206. doi: 10.1038/s41467-022-33945-y (PMC9584949; doi:10.1038/s41467-022-33945-y)
Supplement: Supplementary file 4 — Description of Additional Supplementary Files [file 41467_2022_33945_MOESM4_ESM.docx]

**Description of Additional Supplementary Files**

File Name: Supplementary Data 1

Description: List of samples diagnosed with ESCC from TCGA ESCA project

File Name: Supplementary Data 2

Description: Variant allele frequency of TP53 mutants in normal human esophageal epithelium (EE)

File Name: Supplementary Data 3

Description: Distribution of basal cell size of YFP and p53*/wt clones

File Name: Supplementary Data 4

Description: Proportion of labelled projected area in induced YFP mice

File Name: Supplementary Data 5

Description: Proportion of labelled projected area in induced p53*/wt mice

File Name: Supplementary Data 6

Description: Density of p53*/wt clones over the time

File Name: Supplementary Data 7

Description: Quantification of EdU-labelled basal cells in p53*/wt clones and non-labelled (p53wt/wt) areas

File Name: Supplementary Data 8

Description: in vitro cell competition assay

File Name: Supplementary Data 9

Description: RNAseq Transcripts Per Million

File Name: Supplementary Data 10

Description: Differentially expressed genes identified from RNAseq

File Name: Supplementary Data 11

Description: RNAseq: Gene Ontology analysis

File Name: Supplementary Data 12

Description: Proportion of labelled projected area in induced p53*/- mice

File Name: Supplementary Data 13

Description: Number of cells with ≥ double sized nucleus in p53 mutant EE

File Name: Supplementary Data 14

Description: Targeted sequencing data from control and DEN-treated samples

File Name: Supplementary Data 15

Description: dN/dS results from DEN-treated and control mouse EE

File Name: Supplementary Data 16

Description: Labelled p53*/wt clone area in control and DEN-treated EE

File Name: Supplementary Data 17

Description: Size distribution of GFP ± EE lesions in DEN-treated mice

File Name: Supplementary Data 18

Description: Number of EE lesions found in control and DEN-treated mice

File Name: Supplementary Data 19

Description: Size distribution of EE lesions in DEN-treated p53wt/wt and p53*/wtmice

File Name: Supplementary Data 20

Description: Quantification of centrosomes

File Name: Supplementary Data 21

Description: Pathology result of macroscopic tumors

File Name: Supplementary Data 22

Description: Targeted sequencing data from frozen section of tumors

File Name: Supplementary Data 23

Description: dN/dS results from sequencing of macroscopic tumors

File Name: Supplementary Data 24

Description: Low input targeted sequencing data from DEN-treated p53wt/wt and p53*/wt EE

File Name: Supplementary Data 25

Description: Number of p53*/- and p53wt/-tumors

File Name: Supplementary Data 26

Description: Low input targeted sequencing data from DEN-treated p53 mutant LOH model mice
